# Innovative utilization of cell membrane‐coated nanoparticles in precision cancer therapy

**DOI:** 10.1002/EXP.20230164

**Published:** 2024-03-21

**Authors:** Yiling He, Shuquan Zhang, Yaoguang She, Zhaoshan Liu, Yalan Zhu, Qinzhen Cheng, Xiaoyuan Ji

**Affiliations:** ^1^ Department of Pharmacy Jinhua Municipal Central Hospital Jinhua Zhejiang China; ^2^ Department of Orthopedics Integrated Chinese and Western Medicine Hospital Tianjin University Tianjin China; ^3^ Department of Orthopedics Tianjin Nankai Hospital Tianjin China; ^4^ Department of Orthopedics Tianjin Hospital of Integrated Chinese and Western Medicine Tianjin China; ^5^ Department of General Surgery First Medical Center Chinese People's Liberation Army (PLA) General Hospital Beijing China; ^6^ Laboratory of Immune Cell Biology Center for Cancer Research National Cancer Institute National Institutes of Health Bethesda Maryland USA; ^7^ Academy of Medical Engineering and Translational Medicine Medical College Tianjin University Tianjin China

**Keywords:** cancer therapy, cell membrane‐coated nanoparticles, cell membrane‐coated nanoparticles, nanomaterials, targeted drug delivery

## Abstract

Cell membrane‐coated nanoparticles (CMNPs) have recently emerged as a promising platform for cancer therapy. By encapsulating therapeutic agents within a cell membrane‐derived coating, these nanoparticles combine the advantages of synthetic nanoparticles and natural cell membranes. This review provides a comprehensive overview of the recent advancements in utilizing CMNPs as effective drug delivery vehicles for cancer therapy. The synthesis and fabrication methods of CMNPs are comprehensively discussed. Various techniques, such as extrusion, sonication, and self‐assembly, are employed to coat synthetic nanoparticles with cell membranes derived from different cell types. The cell membrane coating enables biocompatibility, reducing the risk of an immune response and enhancing the stability of the nanoparticles in the bloodstream. Moreover, functionalization strategies for CMNPs, primarily chemical modification, genetic engineering, and external stimuli, are highlighted. The presence of specific cell surface markers on the coated membrane allows targeted drug delivery to cancer cells and maximizes therapeutic efficacy. Preclinical studies utilizing CMNPs for cancer therapy demonstrated the successful delivery of various therapeutic agents, such as chemotherapeutic drugs, nucleic acids, and immunotherapeutic agents, using CMNPs. Furthermore, the article explores the future directions and challenges of this technology while offering insights into its clinical potential.

## Graphical abstract

Cell membrane‐coated nanoparticles (CMNPs) have recently emerged as a promising platform for cancer therapy. This review provides a comprehensive overview of the recent advancements in utilizing CMNPs as effective drug delivery vehicles for cancer therapy, including synthesis methods, functionalization strategies, preclinical studies utilizing CMNPs for cancer therapy, and future directions and challenges of clinical potential of CMNPs.

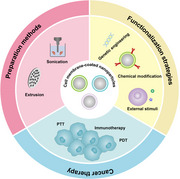

## 1 INTRODUCTION

Cancer treatment remains a significant global health challenge, necessitating the development of innovative therapeutic approaches.^[^ ^1^ ^]^ In recent years, nanotechnology has revolutionized the field of cancer therapy by offering precise and targeted drug delivery systems.^[^ ^2^ ^]^ Among the various nanoscale delivery systems, cell membrane‐coated nanoparticles (CMNPs) have garnered considerable attention for their distinct properties and capabilities in cancer therapy.^[^ ^3^ ^]^ CMNPs are a type of nanomaterial that combines the properties of both cell membranes and nanoparticles. They are created by coating the surface of nanoparticles with a layer of cell membrane derived from a specific cell type. By coating nanoparticles with cell membranes, researchers can take advantage of the unique characteristics of these membranes, such as their surface receptors, adhesion molecules, and immune evasion properties.^[^ ^4^ ^]^ This allows nanoparticles to effectively evade the immune system, prolong their circulation time, and specifically target cancer cells. CMNPs can be derived from various cell sources, including red blood cells (RBCs), immune cells, cancer cells, and platelets.^[^ ^5^ ^]^ Each cell type offers distinct advantages and functionalities that can be harnessed for targeted drug delivery.^[^ ^6^ ^]^ The functionalization of CMNPs further enhances their ability to treat cancer. By attaching targeting ligands, such as antibodies or peptides, to CMNPs, their specificity toward cancer cells or tumor tissues can be improved.^[^ ^7^ ^]^

CMNPs have shown wide‐ranging applications in cancer therapy. In this review, we explore recent advancements in the development of CMNPs for cancer therapy. We discuss the various preparation methods of CMNPs, including direct membrane extraction and coating nanoparticles with cell membranes. These methods allow for the integration of cell membrane components onto the nanoparticle surface, providing enhanced biocompatibility and stability. We also explored the functionalization of CMNPs through the use of specific ligands or antibodies that enable targeted delivery to cancer cells by recognizing specific receptors on their surface. This targeted approach improves the efficacy of drug delivery and reduces off‐target effects. Furthermore, we explored the diverse applications of CMNPs in cancer therapy. The cell membrane coating of CMNPs offers improved biocompatibility, which allows for prolonged circulation time and increased accumulation at the tumor site. Moreover, we emphasize the challenges encountered in this field, such as the reproducibility and scalability of CMNP synthesis, long‐term stability, immunogenicity, and understanding of the pharmacokinetics and biodistribution of CMNPs in vivo. Addressing these challenges is crucial for the successful translation of CMNPs into clinical applications.

## 2 PREPARATION METHODS FOR CMNPS

**FIGURE 1:**
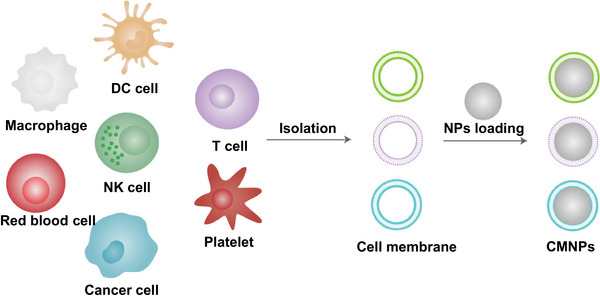
General preparation process for cell membrane‐coated nanoparticles (CMNPs).

The preparation of CMNPs involves the isolation of cell membranes from specific cell sources and their subsequent coating onto synthetic nanoparticles (Figure 1). The choice of cell source is crucial because it determines the properties and functionalities of the resulting nanoparticles. Common cell sources include red blood cells (RBCs), cancer cells, immune cells, and platelets. The extraction of cell membranes can be achieved through various methods, including extrusion, sonication or freeze‒thaw cycles.^[^ ^5^, ^8^ ^]^ After obtaining the cell membranes, various methods, including extrusion, sonication, and electrostatic interactions, have been employed for the successful fusion of cell membranes onto nanoparticles.^[^ ^4^, ^9^ ^]^ This section discusses the different preparation techniques, their advantages, and their limitations.

### 2.1 Isolation of cell membranes

The preparation of CMNPs involves a multiple steps that begin with the isolation of cell membranes from specific cell sources. Various methods can be employed to extract cell membranes.^[^ ^10^ ^]^

One commonly used method is membrane extrusion, in which cell membranes are mechanically disrupted and extruded through small pores to generate nanosized vesicles.^[^ ^11^ ^]^ This method offers control over the size and uniformity of the resulting nanoparticles. However, membrane extrusion can lead to membrane damage or the loss of specific membrane components, which may affect the functionality of the coated nanoparticles.

Sonication is another method used for membrane extraction.^[^ ^12^ ^]^ It involves the use of high‐frequency sound waves to disrupt cell membranes and release cellular contents. Sonication is a rapid and efficient method that provides good yields of cell membranes.^[^ ^13^ ^]^ However, the mechanical forces generated during sonication can potentially damage or fragment membranes, affecting their integrity and functionality. Care should be taken to optimize sonication parameters to minimize membrane damage.

Freeze‒thaw cycles offer a simple and cost‐effective method for membrane extraction. This technique relies on the principle that the formation of ice crystals during freezing can cause mechanical disruption of cell membranes.^[^ ^14^ ^]^ The freeze‒thaw cycles are relatively gentle, preserving the integrity of the membranes. However, repeated freeze‒thaw cycles can potentially lead to the loss of membrane integrity.

In summary, the choice of membrane extraction method depends on various factors, including the desired purity, yield, integrity, and functionality of the extracted membranes. Researchers should carefully consider these factors and select the most suitable method for their specific application.

### 2.2 Coating cell membranes onto nanoparticles

Coating synthetic nanoparticles with cell membranes involves various techniques, each with its own advantages and limitations.^[^ ^15^ ^]^ Extrusion involves coextruding cell membranes and nanoparticles through a porous membrane or microfluidic device. The pressure applied during extrusion facilitates the fusion of cell membranes onto the nanoparticle surface. This scalable approach allows for the incorporation of additional functional components. However, it is important to note that the size and morphology of the resulting coated nanoparticles may be influenced by extrusion conditions.

Sonication, on the other hand, relies on subjecting cell membranes and nanoparticles to ultrasonic waves, inducing membrane fusion onto the nanoparticle surface.^[^ ^16^ ^]^ This method is known for its rapidity and efficiency and can be easily scaled up. However, the high energy input during sonication may damage cell membranes or lead to inconsistent coating quality.

Electrostatic interactions exploit the attractive forces between cell membranes and nanoparticles to facilitate fusion.^[^ ^6^ ^]^ By adjusting the surface charge of the nanoparticles and membrane proteins, a stable and uniform coating can be achieved. However, optimization is often needed to ensure efficient membrane‐nanoparticle binding and prevent premature dissociation.^[^ ^17^ ^]^

In summary, the preparation of CMNPs entails isolating cell membranes from specific sources and applying them to synthetic nanoparticles through various techniques. Each method has distinct advantages and limitations, and the selection of a technique depends on the desired properties and intended applications of the resulting nanoparticles. Ongoing research in this field aims to refine these preparation techniques and explore the expanded potential of CMNPs in cancer therapy.

## 3 FUNCTIONALIZATION OF CELL MEMBRANE‐COATED NANOPARTICLES

**FIGURE 2:**
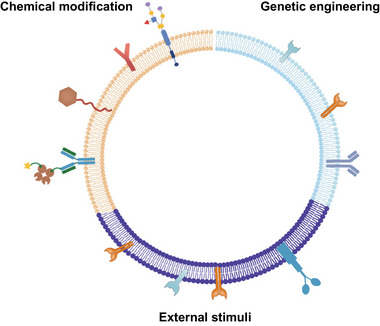
Cell membrane‐coated nanoparticles (CMNPs) can be functionalized via chemical modification, genetic engineering or external stimuli.

CMNPs have gained significant attention in the field of nanomedicine due to their unique properties and potential applications in various therapeutic areas. A pivotal aspect of CMNPs lies in their functionalization, which entails the integration of supplementary functionalities onto the cell membrane coating or the nanoparticle core. The functionalization strategies for CMNPs primarily include chemical modification, genetic engineering, and external stimuli (Figure 2).^[^ ^18^ ^]^ This process enhances the versatility and efficacy of CMNPs, enabling their utilization in diverse therapeutic areas. Summary of the key benefits and drawbacks associated with each functionalization strategy for CMNPs (Table 1).

**TABLE 1:** Overview of functionalization strategies for cell membrane‐coated nanoparticles (CMNPs).

Strategy	Principle	Advantages	Disadvantages
Chemical Modification	Involves altering the surface properties of CMNPs through chemical reactions, allowing for the attachment of specific functional groups or molecules to enhance functionalities.	Precise, Versatile, Established	Nonspecific, Potentially toxic
Genetic Engineering	Uses genetic manipulation techniques to modify the cell membrane, enabling the expression of specific proteins, receptors, or signaling molecules to impart desired functionalities.	Specific, Tailored, Natural	Complex, Regulatory concerns
External Stimuli	Involves the use of external triggers such as light, magnetic fields, temperature, or pH changes to induce controlled release, activation, or modulation of CMNP properties.	Controlled, On‐demand, Minimally invasive	Environmental dependencies, Implementation challenges

### 3.1 Chemical modification

Chemical modification is a widely employed technique for the functionalization of CMNPs. This method involves the use of reactive chemical groups on CMNPs and functional molecules to form covalent or noncovalent bonds, thereby attaching the desired functionalities onto the nanoparticle surface.^[^ ^19^ ^]^ Chemical modification offers precise control over the type and density of functional molecules on CMNPs, allowing tailored modifications to meet specific therapeutic requirements. Several chemical modification strategies are commonly utilized for functionalizing CMNPs.

**FIGURE 3:**
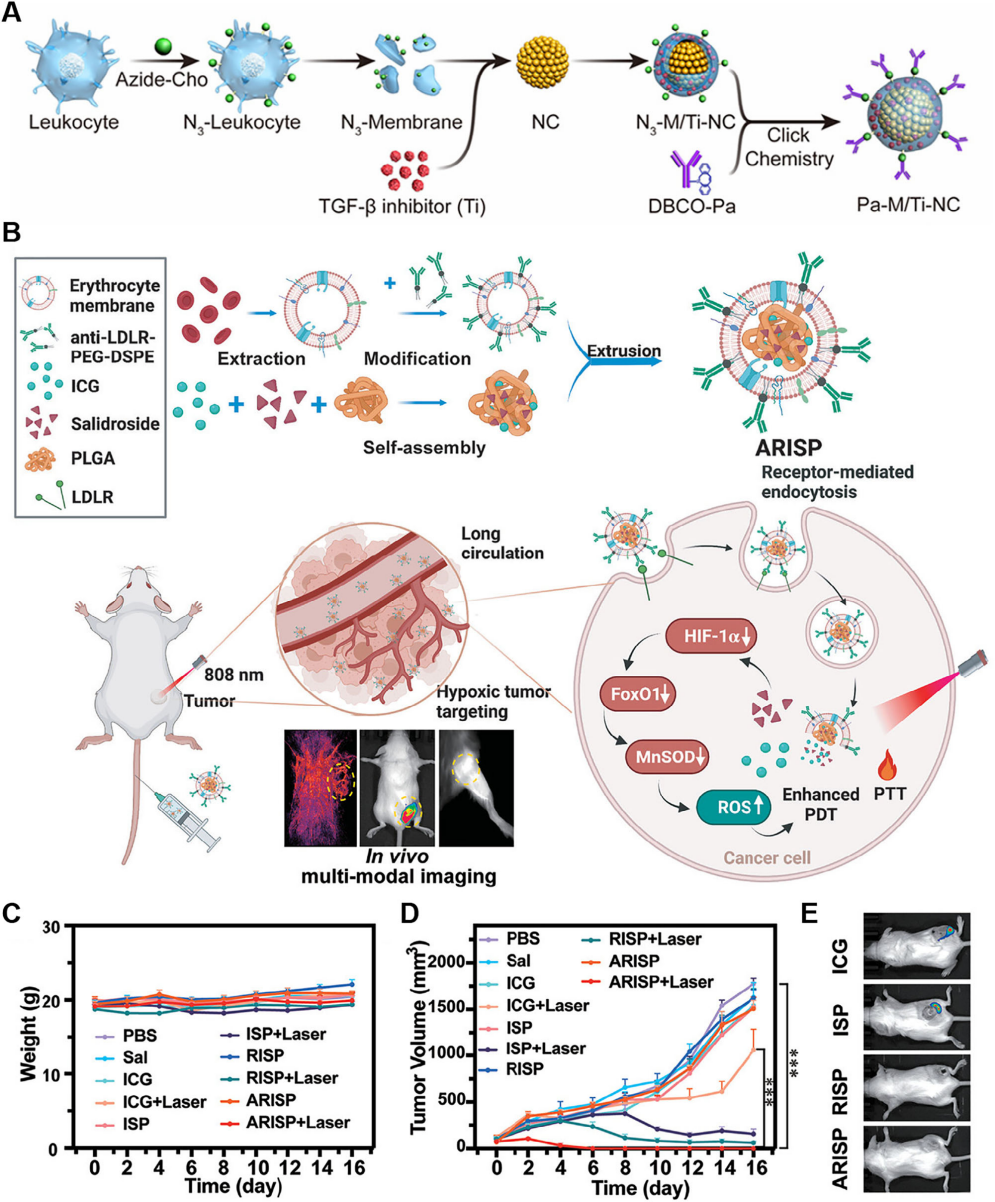
Chemical modification of cell membrane‐coated nanoparticles (CMNPs). (A) Preparation of Pa‐M/Ti‐NCs. Reproduced with permission.^[^ ^20^ ^]^ Copyright 2019, American Chemical Society. (B) Preparation and anticancer mechanism of ARISP. (C) Weight change curves of the mice that received different treatments. (D) Tumor growth curves after different treatments (*n* = 5). (E) Bioluminescence images of representative mice that received different treatments. Reproduced with permission.^[^ ^21^ ^]^ Copyright 2022, Wiley‐VCH.

Click chemistry is a chemical modification method commonly used to modify CMNPs. It is an efficient and specific reaction that can be carried out under mild conditions and can form stable covalent bonds. The azide–alkyne reaction is one of the most common click chemistry reactions and is also known as the azide–alkyne cycloaddition reaction. Through click chemistry reactions, various functional groups, such as fluorescent dyes, antibodies, and polymers, can be introduced on the surface of CMNPs. For instance, Xie et al. synthesized Fe_3_O_4_ magnetic nanoclusters (NCs) as the core of a magnetosome, which exhibited satisfactory superparamagnetism and magnetic control. These NCs served as a platform for Fe accommodation, magnetic resonance imaging (MRI) guidance, and magnetic targeting. To enhance their performance, they coated the NCs with a leukocyte membrane pre‐engineered with azide (N3). This combination not only prolonged circulation time but also facilitated the loading of the hydrophobic TGF‐β inhibitor (Ti) into the membrane and the conjugation of the dibenzocyclooctyne (DBCO)‐modified PD‐1 antibody (Pa) through mild and efficient click chemistry (Figure 3A).^[^ ^20^ ^]^ This article presents a clever design utilizing not only chemical modification strategies but also the incorporation of leukocyte J774A.1 cells as the source of the cell membrane. Research on the stealth properties, biological compatibility, targeting abilities, and tumor microenvironment interactions has been conducted. Despite the complexity of the experimental procedures, the research results demonstrate significant therapeutic efficacy, offering valuable insights for future studies.

Hydrophobic molecules or materials can be attached to nanoparticles through hydrophobic interactions. This approach is particularly useful for modifying nanoparticles with lipids or hydrophobic polymers. The hydrophobic regions of the molecules or polymers interact with the hydrophobic regions on the CMNP surface, leading to surface modification. Cai et al. developed and synthesized a novel nanoplatform known as ARISP. The core of this nanoplatform, ICG‐Sal‐PLGA (ISP), was formed by coloading indocyanine green (ICG) and salidroside (Sal) with poly(lactic‐*co*‐glycolic acid) (PLGA) through emulsification. Moreover, fresh red blood cell membranes (RBCm) were extracted and subjected to overnight coincubation with anti‐low‐density lipoprotein receptor (LDLR)‐PEG2000‐DSPE to facilitate the modification of RBCm with anti‐LDLR. Subsequently, the anti‐LDLR‐modified RBCms were coextruded with the ISP core to create a membrane‐coating nanoparticle, referred to as ARISP (Figure 3B). Throughout the experiment, both body weight and tumor volume were closely monitored. No noticeable changes in body weight were observed (Figure 3C). Notably, treatment of tumors with ARISP plus laser irradiation had an exceptional anticancer effect, resulting in ideal elimination (Figure 3D,E).^[^ ^21^ ^]^ The use of RBCms as a delivery platform for targeted therapy aimed at LDLRs represents a promising and innovative approach. This design not only capitalizes on specific cellular markers for targeted delivery but also harnesses the potential of multimodal imaging to guide and optimize therapeutic outcomes. Overall, this approach represents a sophisticated and multifaceted strategy for addressing tumors, potentially offering enhanced treatment efficacy and precision.

### 3.2 Genetic engineering

Genetic engineering can indeed be used to modify CMNPs. This approach combines the unique properties of both cell membranes and nanoparticles to create a versatile platform for various applications, including drug delivery and therapeutics.^[^ ^22^ ^]^ Genetic engineering can be employed to modify the cell membrane before it is fused with nanoparticles. This can be done by introducing specific genes or genetic modifications into the cells from which the membrane is derived. These modifications can confer additional functionalities to the resulting cell membrane‐coated nanoparticles.^[^ ^23^ ^]^

**FIGURE 4:**
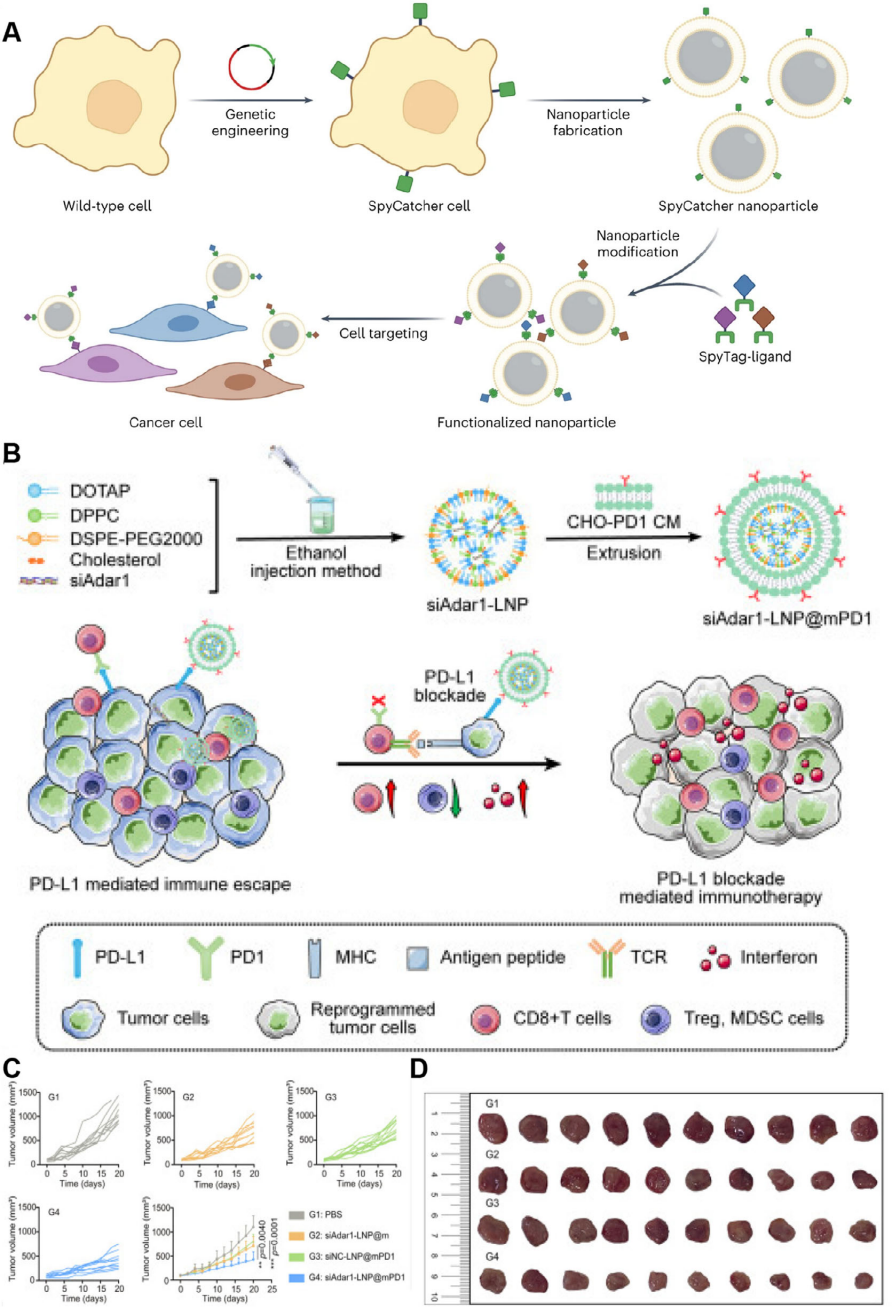
Genetic engineering of cell membrane‐coated nanoparticles (CMNPs). (A) Preparation of functionalized nanoparticles. Reproduced with permission.^[^ ^24^ ^]^ Copyright 2023, Springer Nature. (B) Preparation and anticancer mechanism of siAdar1‐LNP@mPD1. (C,D) Tumor growth kinetics and photographs after receiving different treatments (*n* = 10). Reproduced with permission.^[^ ^25^ ^]^ Copyright 2023, The American Society of Gene and Cell Therapy.

The manipulation of surface expression for naturally occurring membrane‐binding proteins is a relatively straightforward process. However, genetic engineering of soluble proteins on the cell membrane is highly challenging. This process is typically accomplished by fusing a ligand with the transmembrane domain of another protein to anchor it to the cell surface. The resultant hybrid proteins need to be fine‐tuned for particular scenarios and could be susceptible to misfolding, diminished functionality caused by steric hindrance, and limited levels of expression. Zhang et al. devised a versatile CMNP system for manipulating the cellular membrane to express SpyCatcher and utilized it as a tether. Upon encapsulating the modified membrane onto the nanoparticle core, the resultant core nanoparticle formulation can be customized with ligands labeled with SpyTag (Figure 4A). In a murine tumor model, effective targeting and suppression of tumor growth were accomplished. This study showcases the efficacy of modular design principles in expediting the development of CMNP platforms, enabling the incorporation of valuable features without requiring extensive engineering efforts.^[^ ^24^ ^]^

Similarly, Liu et al. developed a genetically engineered nanosystem called siAdar1‐LNP@mPD1 (Figure 4B). This nanosystem was designed to overcome tumor resistance to immune checkpoint blockade (ICB) therapy. This nanosystem consists of a lipid nanoparticle (LNP) that encapsulates small interfering RNA against ADAR1 (siAdar1). ADAR1 is an enzyme involved in adenosine‐to‐inosine (A‐to‐I) RNA editing and has been identified as an intrinsic immunosuppressor associated with resistance to ICB therapy. To enhance therapeutic efficacy, LNPs are enveloped with a plasma membrane derived from genetically modified Chinese hamster ovary (CHO) cells that overexpress PD‐1, a protein involved in immune regulation. The siAdar1‐LNP@mPD1 nanosystem functions by blocking the PD‐1/PD‐L1 signaling axis through the presentation of the PD‐1 protein on the coating membrane. Additionally, the nanosystem efficiently delivered siAdar1 to cancer cells, leading to the silencing of ADAR1 expression. The combined effects of siAdar1‐LNP@mPD1 confer potent and comprehensive antitumor immune responses. These findings underscore the capacity of siAdar1‐LNP@mPD1 to remodel the tumor microenvironment (TME) and enhance the immune response against cancer (Figure 4C,D).^[^ ^25^ ^]^ The selection of CHO cells for genetic expression of PD‐1 in this study stems from their wide application in biopharmaceutical research and production. CHO cells are favored due to their ease of cultivation, stable expression of exogenous proteins, and ability to produce proteins with posttranslational modifications akin to those found in humans. This choice ensures that the murine models used in the study maintain stable and biologically relevant expression of PD‐1, thus better recapitulating human immune responses. Additionally, leveraging CHO cells for PD‐1 expression provides a platform for robust and reliable assessment of antitumor immunotherapies, including the evaluation of PD‐1 inhibitors and the development of novel immunotherapeutic strategies. Overall, the use of CHO cells in this context not only ensures the biological relevance of the model but also facilitates the translation of findings to potential therapeutic interventions, thereby enhancing the clinical significance of the research.

### 3.3 External stimuli

One approach to achieve the functionalization of CMNPs involves the overexpression of specific membrane proteins in response to external stimuli.^[^ ^26^ ^]^ This strategy allows for the incorporation of additional functionalities onto the CMNPs, thereby enhancing their therapeutic potential and enabling targeted drug delivery. By utilizing external stimuli, the expression of membrane proteins can be selectively upregulated. These membrane proteins can be engineered to possess desired functionalities, such as targeting ligands or receptors. Upon exposure to the appropriate stimulus, the membrane proteins are overexpressed on the CMNPs, leading to functionalization of the nanoparticle surface.

**FIGURE 5:**
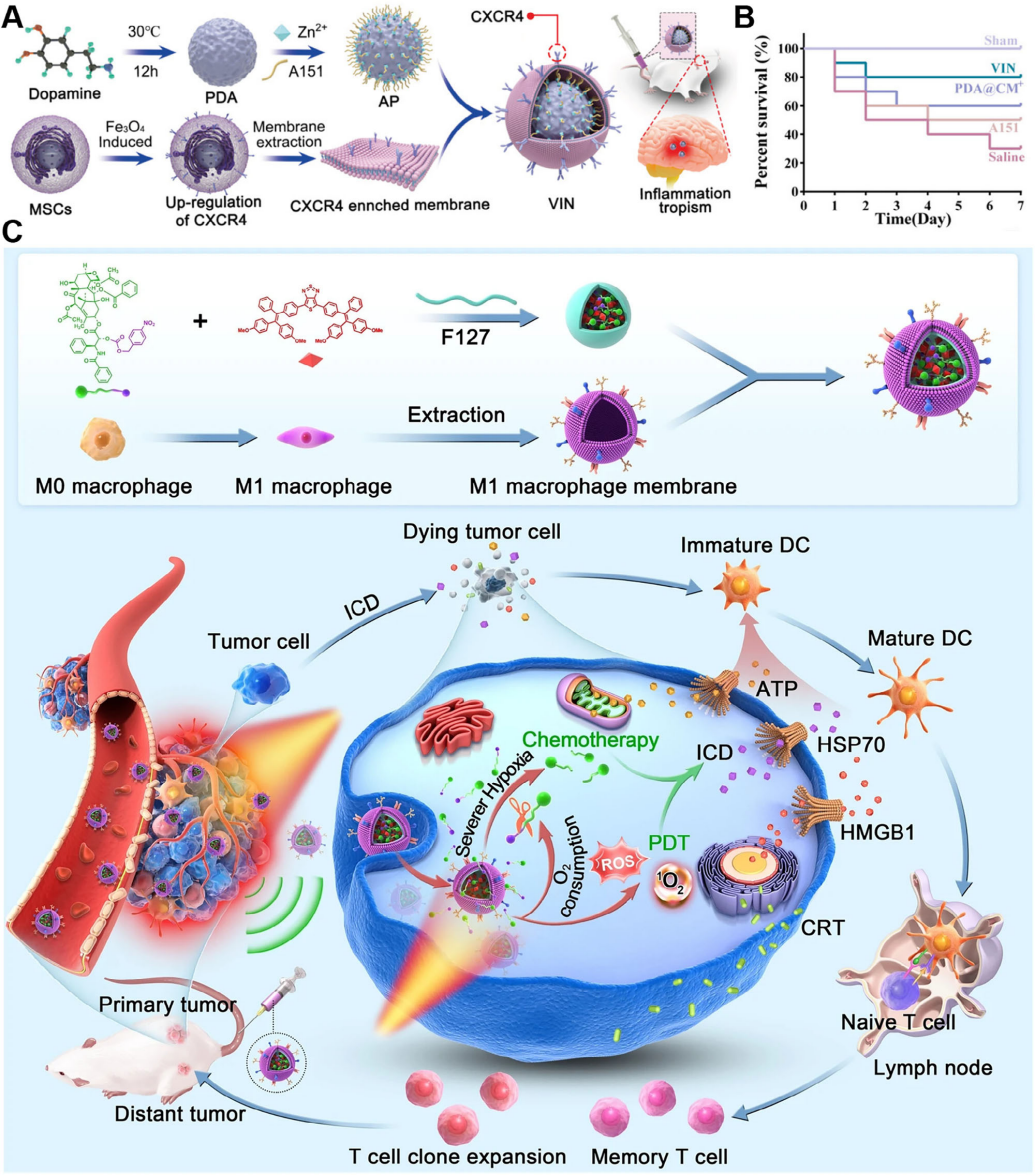
External stimuli for cell membrane‐coated nanoparticles (CMNPs). (A) Preparation of VIN. (B) Survival rate of rats treated with different agents (*n* = 10). Reproduced with permission.^[^ ^27^ ^]^ Copyright 2021, Wiley‐VCH. (C) Preparation and anticancer mechanism of M1‐MPNPs. Reproduced with permission.^[^ ^28^ ^]^ Copyright 2023, Springer Nature.

Compared to chemical modification and genetic engineering techniques, the modification of CMNPs using external stimuli is generally simpler and more straightforward. For example, mesenchymal stem cells (MSCs) are modified to upregulate C‐X‐C chemokine receptor type 4 (CXCR4) in response to Fe3O4 stimulation. Specifically, Zhang et al. reported the development of an engineered C‐X‐C chemokine ligand 12 (CXCL12) biomimetic decoy‐integrated versatile immunosuppressive nanoparticle (VIN) for managing the overactivated brain immune microenvironment. VIN is prepared by coating membrane vesicles derived from mesenchymal stem cells (MSCs) enriched with CXCR4, which is facilitated by the stimulation of Fe_3_O_4_, onto polydopamine nanospheres (PDA) that are loaded with A151, a pharmacological agent with immunosuppressive properties (Figure 5A). In an in vivo experimental study, VIN demonstrated excellent therapeutic effects (Figure 5B).^[^ ^27^ ^]^

Tang et al. introduced a self‐accelerated nanoplatform that combines an aggregation‐induced emission luminogen (AIEgen) and a hypoxia‐responsive prodrug for multifunctional image‐guided combination immunotherapy. To formulate a high‐performance therapeutic agent, AIEgen was combined with a hypoxia‐responsive paclitaxel (PTX) prodrug and encapsulated in nanoparticles. These nanoparticles were further camouflaged with the M1 macrophage membrane obtained by stimulating RAW264.7 cells with external stimuli to induce M1 polarization (Figure 5C). Consequently, the synergistic application of photodynamic therapy (PDT) and PTX chemotherapy leads to the induction of immunogenic cell death in cancer cells. This process not only stimulates a potent antitumor immune response to suppress the primary tumor but also effectively inhibits the growth of distant tumors in female mice bearing 4T1 tumors.^[^ ^28^ ^]^ By utilizing external stimuli to induce M1 polarization in macrophages, the inherent antitumor properties of M1 macrophages can be harnessed. Extracting cell membranes for nanoparticle encapsulation capitalizes on the unique surface markers and functional components present in the membranes, offering a targeted and immunologically relevant delivery system. This strategy not only facilitates the design of tumor‐targeted drug delivery systems but also leverages the inherent immunomodulatory functions of M1 macrophages to enhance antitumor efficacy.

## 4 CMNPS DERIVED FROM VARIOUS CELL SOURCES IN CANCER THERAPY

CMNPs have emerged as a highly promising approach in the field of cancer therapy (Table 2). The unique properties and versatile applications of these materials make them valuable tools for drug delivery, imaging, and combination therapy. In this section, we will delve into the applications of CMNPs derived from different cell sources, including RBCs, immune cells, cancer cells, and platelets, and explore how they contribute to improving cancer treatment outcomes.

**TABLE 2:** Examples of CMNPs employed in cancer therapy.

Source cells	Core nanoparticles	Engineering method	Diseases	Refs.
RBC	ZIF‐8‐DOX‐LY	Primitive membrane	Breast cancer	[33]
	PTX/CMCS NPs	Chemical modification	Epithelial cancer	[34]
	BPtI	Chemical modification	Melanoma	[35a]
	ALA/PAMAM	Chemical modification	Melanoma	[35b]
	HDC	Chemical modification	Breast cancer	[36]
Macrophages	NGs	Primitive membrane	Breast cancer, pancreatic cancer, colon cancer	[44]
	anti‐PD‐L1 and CXCL10	Chemical modification	Glioblastoma multiforme	[45]
	PLGA/RAPA	Genetic engineering	Glioblastoma multiforme	[46]
	CPI‐444	Genetic engineering	Melanoma	[47]
T‐cell	DON	Genetic engineering	Lung adenocarcinoma	[53]
	ORY‐1001‐loaded BSA core	Genetic engineering	Breast cancer, colon cancer, melanoma	[54]
	AIE NPs	Genetic engineering	Glioblastoma multiforme	[55]
NK cell	T‐NPs	Primitive membrane	Breast cancer	[56]
	AsHMS‐TA/Fe^III^	Primitive membrane	Hepatocellular carcinoma	[60]
	AIEdots	Primitive membrane	Glioblastoma multiforme	[61]
	DOX	Membrane fusion	Breast cancer	[58]
DC	MPLA	Membrane fusion	Squamous cell carcinoma	[64]
	/	Genetic engineering	Breast cancer, colon cancer, melanoma	[63b]
	/	Genetic engineering	Melanomas, lung carcinoma	[65]
Cancer cell	PCEC@BTZ nanoparticles	Primitive membrane	Multiple myeloma	[73]
	GINPs	Membrane fusion	Glioma	[74]
	NPs@G	Membrane fusion	Glioblastoma multiforme	[75]
Platelet	L/DP&PPa	Primitive membrane	Breast cancer	[80]
	HGNs	Primitive membrane	Breast cancer	[81]
	Fe_3_O_4_‐SAS	Primitive membrane	Breast cancer	[82]

### 4.1 Red blood cell (RBC)‐derived membrane‐coated nanoparticles

**FIGURE 6:**
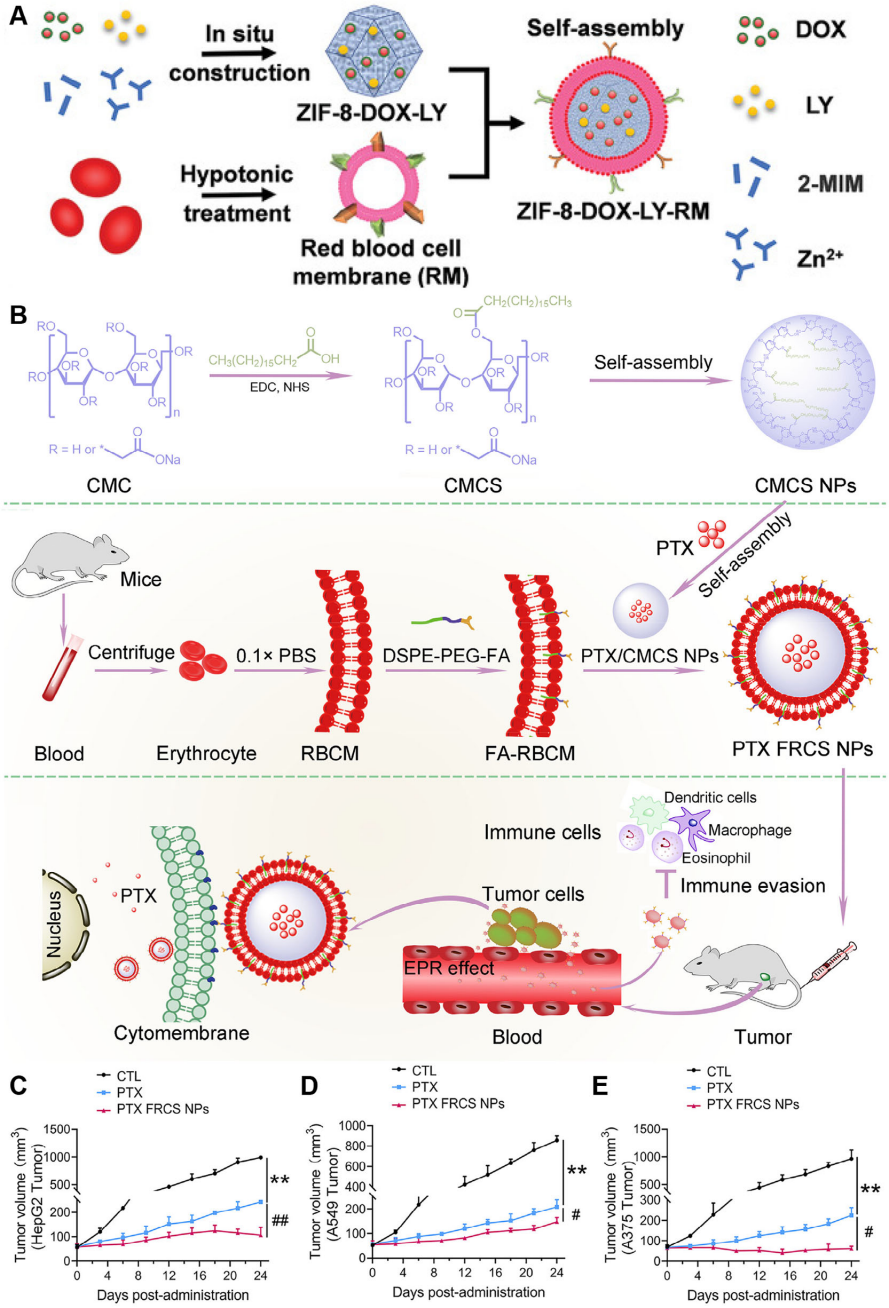
Red blood cell‐derived membrane‐coated nanoparticles (RBC‐NPs) for cancer therapy. (A) Preparation of ZIF‐8‐DOX‐LY‐RM. Reproduced with permission.^[^ ^33^ ^]^ Copyright 2021, Wiley‐VCH. (B) Preparation and anticancer mechanism of PTX FRCS NPs. Reproduced with permission.^[^ ^33^ ^]^ Copyright 2021, Wiley‐VCH. (C–E) Cancer growth curves of HepG2 cells, A549 cells and A375 cells during therapy. Reproduced with permission.^[^ ^34^ ^]^ Copyright 2022, Elsevier.

In 2011, the emergence of red blood cell membrane‐coated nanoparticles (RBC‐NPs) marked a significant advancement in the field of cell membrane‐enveloped nanoparticles.^[^ ^29^ ^]^ This breakthrough laid the foundation for the development of nanoparticles cloaked with various cell membranes. The utilization of RBC membranes as coatings for nanoparticles offers several advantages in cancer therapy.^[^ ^30^ ^]^ RBC‐derived membranes offer an optimal solution for drug delivery due to their exceptional biocompatibility, flexibility, and presence of distinct surface markers, such as CD47 and glycans. The presence of these surface markers plays a crucial role in reducing the phagocytosis of nanoparticles by immune cells, effectively extending the nanoparticle circulation time within the body.^[^ ^5b^ ^]^ By coating nanoparticles with RBC‐derived membranes, a stealth effect is achieved, enabling the nanoparticles to evade immune system detection.^[^ ^31^ ^]^ Consequently, the nanoparticles exhibit enhanced accumulation within tumor tissues while minimizing systemic toxicity.^[^ ^32^ ^]^ This stealth effect significantly augments the specificity and efficiency of drug delivery to tumors, making RBC‐derived membranes an exceptional choice for targeted therapy. For example, Yang et al. introduced a biomimetic nanodrug termed ZIF‐8‐DOX‐LY‐RM. This nanodrug was coated with red blood cell membrane (RM) and encapsulated in a transforming growth factor β receptor inhibitor (TGFBR1) and doxorubicin (DOX) for efficient chemotherapy (Figure 6A). By harnessing the biomimetic characteristics of the RM, ZIF‐8‐DOX‐LY‐RM can accumulate specifically in tumor tissues, evade immune surveillance, and maintain prolonged circulation in the bloodstream.^[^ ^33^ ^]^

However, RBC‐NPs lack specific targeting ligands and active transmembrane transport mechanisms in tumors. Chemical modification, such as the use of folic acid (FA), is a common approach for addressing this issue, but it may denature functional proteins in RBC‐NPs. To maintain membrane integrity, a nondisruptive functionalization strategy using lipid tethers such as 1,2‐distearoyl‐sn‐glycero‐3‐phosphoethanolamine (DSPE) can naturally insert specific binding molecules into RBC‐NPs. Chen et al. developed a novel nanocarrier system called PTX FRCS NPs, which consists of a “core–shell” architecture. The core of these nanoparticles is composed of PTX encapsulated with polymer CMCS NPs based on stearic acid (SA) and carboxymethyl cellulose (CMC). The shell of the nanoparticles is formed by cloaking them with red blood cell membranes (RBCMs) modified with DSPE‐ polyethylene glycol (PEG)‐FA (Figure 6B). This innovative design allows targeted drug delivery. PTX FRCS NPs exhibited excellent biocompatibility and sensitivity to acidic TMEs. In terms of functionality, the FRCS NPs significantly enhanced the therapeutic efficacy of PTX against various epithelial malignancies, such as hepatocellular carcinoma, lung cancer, and malignant melanoma (Figure 6C–E).^[^ ^34^ ^]^ In addition to modifying FAs, RBCMs can also be decorated with other targeting ligands, such as RGD (Arg‐Gly‐Asp) and AS1411.^[^ ^35^ ^]^ These targeting ligands can be modified through a similar approach to DSPE‐PEG‐FA by incorporating them into RBCMs, thereby imparting nanoparticles with cell recognition and targeting capabilities.

Moreover, RBC‐NPs can be functionalized with a photosensitizer to harness their photothermal therapeutic effect and enhance treatment specificity. For instance, Yin et al. utilized docetaxel and calcitriol, which were separately conjugated with heparin sulfate, to synthesize prodrugs. These prodrugs were then mixed together to prepare mixed micelles. Simultaneously, they prepared RBC‐NPs and decorated them with the photosensitizer 1,1‐dioctadecyl‐3,3,3,3‐tetramethylindotricarbocyanine iodide (DiR). The engineered RBC membrane was then applied to cover the surface of the mixed micelles, resulting in the formation of HDC‐DM.^[^ ^36^ ^]^

The utilization of RBC‐NPs in the treatment of tumors has demonstrated encouraging outcomes in preclinical investigations. These nanoparticles have demonstrated improved tumor accumulation, enhanced cellular uptake, and increased therapeutic efficacy compared to free drugs or uncoated nanoparticles. Additionally, RBC‐NPs can be further modified to incorporate targeting ligands or imaging agents, enabling precise tumor targeting and real‐time monitoring of therapeutic response.

### 4.2 Immune cell‐derived membrane‐coated nanoparticles

Immune cell‐derived membrane‐coated nanoparticles have exhibited significant promise in the field of cancer immunotherapy. These nanoparticles can imitate the surface characteristics of immune cells by utilizing membranes sourced from various immune cells, including macrophages, T cells, natural killer cells (NK cells), and dendritic cells (DCs).^[^ ^37^ ^]^ This allows them to mimic the properties of immune cells and effectively interact with the TME.^[^ ^5e^ ^]^ By employing this strategy, the immunomodulatory effects of the nanoparticles increase, facilitating an immune reaction against cancer cells. Moreover, immune cell‐derived membrane coatings can be engineered to include immunomodulatory substances, thereby enhancing the effectiveness of immunotherapy.^[^ ^38^ ^]^

#### 4.2.1 Macrophage‐derived membrane‐coated nanoparticles

The use of macrophage‐derived membrane‐coated nanoparticles (MM‐NPs) is a promising strategy for cancer treatment.^[^ ^39^ ^]^ The use of macrophage membranes in nanoparticle coatings offers several advantages for cancer therapy.^[^ ^40^ ^]^ First, macrophages can infiltrate tumor tissues due to their natural tumor‐homing properties. By utilizing macrophage membranes, MM‐NPs can mimic the surface markers and receptors present on macrophages, allowing improved targeting and accumulation within tumors.^[^ ^41^ ^]^ The membrane coating can camouflage cells by leveraging the macrophage‐derived membrane, which aids in evading immune surveillance and diminishing recognition by the immune system.^[^ ^42^ ^]^ This stealth effect extends the circulation duration of the nanoparticles. Additionally, macrophages play a crucial role in the TME and can interact with tumor cells and other immune cells.^[^ ^43^ ^]^ The incorporation of macrophage membrane proteins onto the surface of MM‐NPs allows specific interactions with tumor cells, facilitating enhanced cellular uptake and intracellular drug delivery.

**FIGURE 7:**
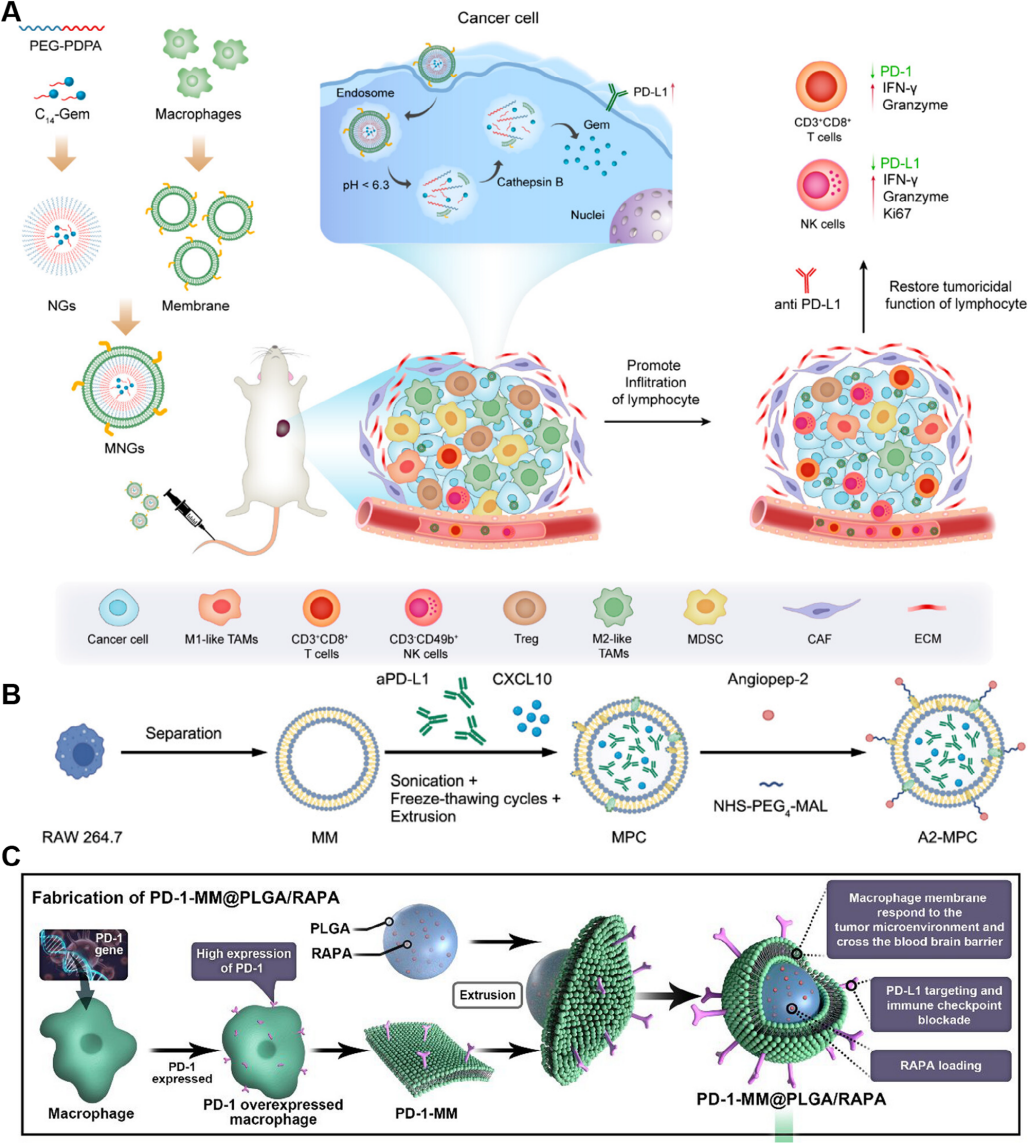
Macrophage‐derived membrane‐coated nanoparticles (MM‐NPs) for cancer therapy. (A) Preparation and anticancer mechanism of MNGs. Reproduced with permission.^[^ ^44^ ^]^ Copyright 2023, American Chemical Society. (B) Preparation of A2‐MPC. Reproduced with permission.^[^ ^45^ ^]^ Copyright 2023, Wiley‐VCH. (C) Preparation of PD‐1‐MM@PLGA/RAPA. Reproduced with permission.^[^ ^46^ ^]^ Copyright 2022, American Chemical Society.

The application of MM‐NPs in cancer therapy has exhibited promising outcomes in preclinical studies. For instance, Zhang et al. conducted a study in which they developed a pH‐responsive drug release system called macrophage membrane‐coated nangemcitabine (MNG), which demonstrated deep intratumor penetration. The primary objective was to increase lymphocyte infiltration and synergize with anti‐programmed death‐ligand 1 (PD‐L1) antibody therapy to restore the tumoricidal effects of cancer immunotherapy. MNGs were created by cloaking the nangemcitabine system (NG) with a macrophage membrane (MM) to mimic the tumor‐targeting features of macrophages (Figure 7A). This approach stems from the tumor‐tropism traits exhibited by macrophages and their capacity to interact with neighboring cells within tumors. Gemcitabine (Gem), a potent chemoimmunomodulatory molecule known for its ability to eliminate immunosuppressive cells and enhance immune recognition, was modified into a cathepsin B‐sensitive hydrophobic prodrug (C14‐Gem) for loading into MNGs to synergize with anti‐PD‐L1 therapy.^[^ ^44^ ^]^

Glioblastoma multiforme (GBM) is a typical immunosuppressive tumor characterized by T‐cell exhaustion, with low levels of CD8^+^ T cells and a limited response from the remaining CD4^+^ T cells against antigen attack. In recent years, biomimetic nanoparticles based on cell membranes have demonstrated remarkable tumor‐targeting capabilities and the potential to modulate the TME. These advancements hold great promise for the development of immunotherapies for GBM. Ju et al. developed a biomimetic nanoparticle named A2‐MPC, which is a biomimetic drug delivery system designed to enhance anti‐PD‐L1 therapy in GBM. RAW 264.7 cells, known for their tumor tropism and blood‐tumor barrier (BTB) penetration ability, were used to prepare the initial macrophage‐membrane (MM)‐camouflaged nanovesicle called MPC. This MPC encapsulates anti‐PD‐L1 and C‐X‐C motif chemokine ligand 10 (CXCL10) using the sonication, freeze–thawing cycles, and extrusion (SFTE) approach. To create A2‐MPCs, the angiopep‐2 peptide and MM were linked through a click reaction using the crosslinker NHS‐PEG4‐MAL. A2‐MPC combines the natural tumor tropism of the macrophage membrane with the angiopep‐2 peptide, enabling specific delivery to GBM cells and efficient BTB penetration. By encapsulating CXCL10 and anti‐PD‐L1, A2‐MPC effectively delivered these proteins to the tumor area (Figure 7B). The angiopep‐2 peptide triggers transcytosis via vesicular trafficking, further enhancing targeted delivery. A2‐MPCs utilizing the macrophage membrane to deliver CXCL10 and anti‐PD‐L1 antibodies are specifically designed to alleviate the immunosuppressive microenvironment of tumors. This innovative approach holds great promise as a strategy for immunotherapy in brain tumors.^[^ ^45^ ^]^

Genetically engineered macrophages modified with specific molecular markers on their membrane surface and loaded with nanoparticles have emerged as a groundbreaking approach for tumor therapy. By utilizing advanced genetic engineering techniques, these macrophages can be precisely engineered to express therapeutic molecules or targeting ligands on their membranes, enhancing their tumor‐specific recognition and interaction. The loaded nanoparticles further augment the therapeutic potential of the NPs by delivering various payloads, such as drugs, genes, or imaging agents, directly to the tumor site. Wang et al. introduced a novel nanoplatform, PD‐1‐MM@PLGA/RAPA, which involves genetic engineering of the macrophage membrane to increase the expression of programmed cell death‐1 (PD‐1) (referred to as PD‐1‐MM). To address the limitations in the bioavailability and bioactivity of rapamycin (RAPA), they employed nanoprecipitation techniques to fabricate poly(lactic‐*co*‐glycolic acid) nanoparticles loaded with RAPA (referred to as PLGA/RAPAs). Finally, they encapsulated PLGA/RAPA nanoparticles within the PD‐1‐MM, resulting in the synthesis of PD‐1‐MM@PLGA/RAPA (Figure 7C).^[^ ^46^ ^]^ Similarly, Zhang et al. developed macrophage cellular vesicles (aPD‐1‐scFv NVs) that display an anti‐PD‐1 single‐chain variable fragment antibody (referred to as aPD‐1‐scFv). The aPD‐1‐scFv NVs were designed to counteract the inhibitory effects of PD‐1 and enhance the activation of T cells against tumors. Furthermore, they incorporated CPI‐444, an antagonist of the A2a adenosine receptor, into aPD‐1‐scFv NVs (CPI‐444‐aPD‐1‐scFv NVs) to enhance the ability of T cells to counteract adenosine, an immunosuppressive metabolite generated by cancer cells. Through the synergistic approach of combining PD‐1 blockade and adenosine antagonism, NVs containing CPI‐444 and aPD‐1‐scFv exhibited substantial increases in both the density and functionality of T cells infiltrating tumors. This direct intervention was proven to be highly effective at suppressing tumor progression and metastasis.^[^ ^47^ ^]^

#### 4.2.2 T‐cell‐derived membrane‐coated nanoparticles

T‐cell‐derived membrane‐coated nanoparticles (TM‐NPs) are designed to mimic the natural cell membrane of T cells and thus provide several advantages for cancer immunotherapy.^[^ ^48^ ^]^ They consist of a nanoparticle core (made of lipids, polymers, or metals) and a T‐cell membrane (TM) shell obtained from donor T cells.^[^ ^49^ ^]^ The TM coating allows for specific targeting of cancer cells by incorporating surface proteins such as T‐cell receptors (TCRs) that recognize cancer cell antigens.^[^ ^50^ ^]^ This targeting enhances therapeutic outcomes and reduces off‐target effects.^[^ ^51^ ^]^ Moreover, the TM coating provides immune evasion capabilities, allowing TM‐NPs to persist in the TME and accumulate at the tumor site. Additionally, the TM coating enhances immune activation by incorporating TM components such as costimulatory molecules and cytokines. This activation promotes robust antitumor immune responses.^[^ ^52^ ^]^

**FIGURE 8:**
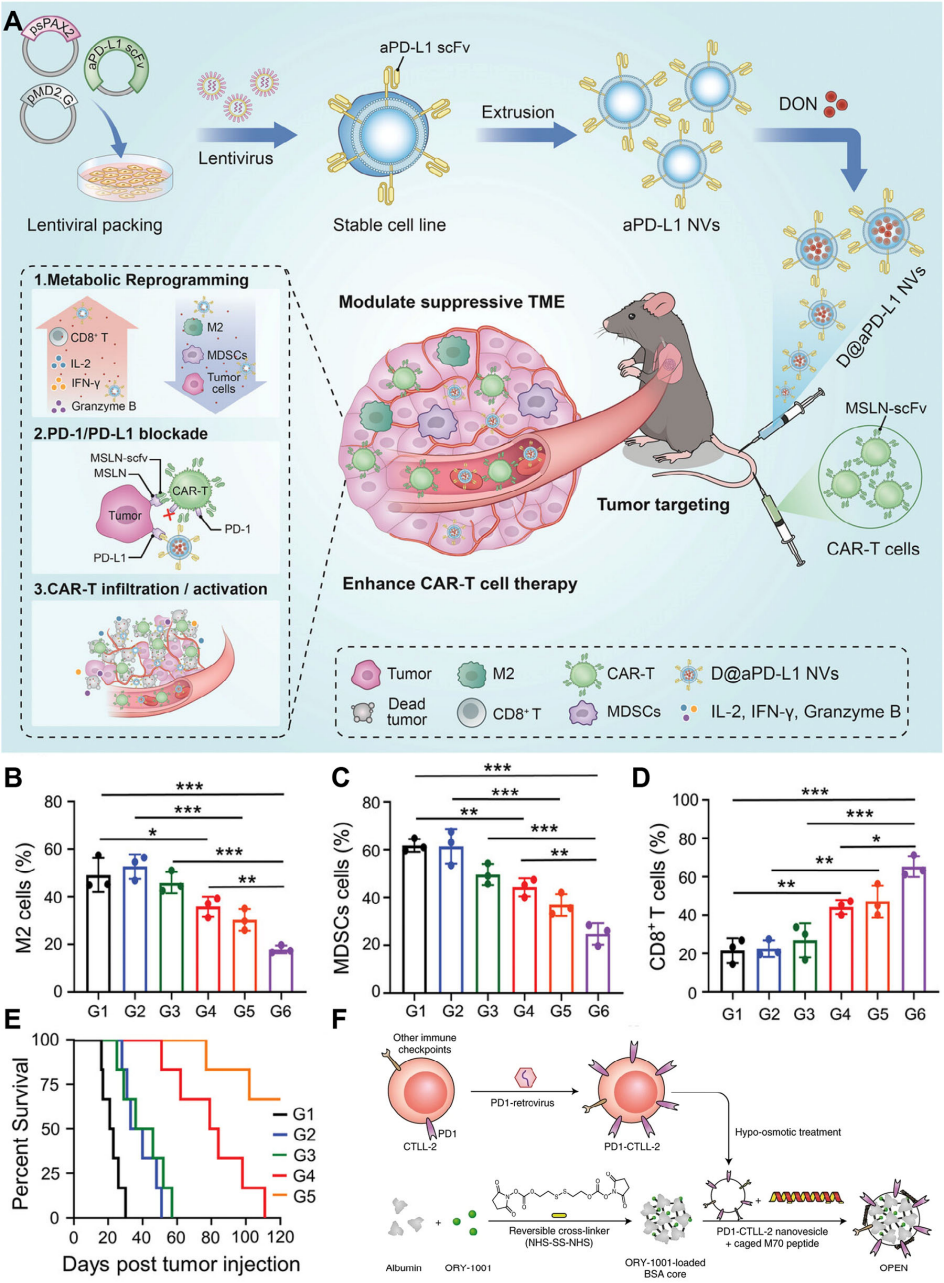
T‐cell‐derived membrane‐coated nanoparticles (TM‐NPs) for cancer therapy. (A) Preparation and anticancer mechanism of D@aPD‐L1 NVs. (B–D) Percentages of M2 macrophages, MDSCs and CD8^+^ T cells that infiltrated tumor tissues. (E) Survival curves of mice after different treatments (*n *= 6). Reproduced with permission.^[^ ^53^ ^]^ Copyright 2023, Wiley‐VCH. (F) Preparation of OPEN. Reproduced with permission.^[^ ^54^ ^]^ Copyright 2021, Springer Nature.

These nanovesicles are engineered to express a high‐affinity anti‐PD‐L1 scFv and are loaded with the glutamine antagonist 6‐diazo‐5‐oxo‐l‐norleucine (DON). The specific binding of the anti‐PD‐L1 scFv to PD‐L1 on tumor cells enables the targeted delivery of DON by the D@aPD‐L1 nanovesicles directly to the tumor site. This innovative approach effectively addresses the issue of PD‐L1 upregulation on tumor cells, which can lead to premature exhaustion of CAR‐T cells. Additionally, D@aPD‐L1 nanovesicles reduced the population of immunosuppressive cells within the TME (Figure 8B,C) and promoted the recruitment of inflammatory cells to tumor tissues (Figure 8D). These unique characteristics of D@aPD‐L1 nanovesicles enhance the infiltration and functional capabilities of CAR‐T cells, ultimately leading to improved overall survival (Figure 8E).^[^ ^53^ ^]^

Immunotherapy has revolutionized cancer treatment by leveraging the immune system to specifically target and eliminate cancer cells. However, increasing intratumoral levels of type I interferons (IFNs) via current methods such as recombinant human IFN injection has limitations in terms of tumor targeting, efficacy, and systemic immune toxicity. Alternative approaches such as chemical drugs, molecular targeted drugs, and epigenetic drugs can induce IFN expression to some extent but lack specificity and have suboptimal effects. Additionally, upregulating intratumoral IFNs can lead to immune checkpoint molecule expression, causing immune evasion and immunosuppression. To address these challenges, Li et al. developed a novel approach. They genetically engineered T cells to overexpress PD‐1 and extracted cell membrane vesicles from these cells. They loaded the potent IFN inducer ORY‐1001 into albumin nanoparticles and coated the engineered TM vesicles with these nanoparticles. Surface modification using pore‐forming activity‐caged macrolittin 70 (cM70) resulted in the formation of epigenetic regulation nanovesicles called OPENs (ORY‐1001‐loaded and PD‐1‐overexpressing T lymphocyte membrane‐decorated epigenetic nanoinducer) (Figure 8F). OPEN nanoparticles offer targeted delivery of IFN inducers. By utilizing PD‐1‐overexpressing T‐cell membranes, they specifically target tumor cells and increase intratumoral IFN levels. The inclusion of ORY‐1001 within the nanovesicles further enhances the therapeutic potential of these nanovesicles. This innovative strategy aims to overcome the limitations of current IFN‐based therapies by maximizing IFN induction efficacy while minimizing systemic immune toxicity. In conclusion, this research has the potential to improve cancer treatment outcomes and contribute to the progress of immunotherapy.^[^ ^54^ ^]^ In addition to their potential in immunotherapy, combining genetically engineered T cells with photothermal therapy (PTT) is a promising strategy for achieving synergistic therapeutic outcomes. For example, Tang et al. successfully created T‐cell‐mimicking nanoparticles, known as aggregation‐induced emission (AIE) nanoparticles, coated with genetically engineered T‐cell membranes (CM@AIE NPs). These innovative CM@AIE NPs effectively induce PTT, resulting in complete inhibition of tumor formation and recurrence.^[^ ^55^ ^]^

Overall, TM‐NPs show immense potential in the field of cancer immunotherapy, primarily because of their capacity to selectively target cancer cells, evade immune detection, and stimulate immune responses. These nanoparticles provide a versatile platform for delivering therapeutic agents and can be customized to suit individual patient requirements. Current research efforts are concentrated on refining the design and manufacturing protocols of TM‐NPs, as well as investigating their synergistic effects when combined with other immunotherapeutic approaches.

#### 4.2.3 NK cell‐derived membrane‐coated nanoparticles

NK cell‐derived membrane‐coated nanoparticles (NK‐NPs) are a promising antitumor therapy.^[^ ^56^ ^]^ NK cells, as vital elements of the innate immune system, play a critical role in initiating an immune response against cancer.^[^ ^57^ ^]^ Coating nanoparticles with NK cell membranes offers several advantages. First, it enables specific targeting of tumor cells by transferring activating receptors from NK cells to coated nanoparticles. This enhances their ability to bind and selectively target tumor cells, improving therapeutic efficacy. Second, the membrane coating provides immune evasion capabilities, allowing the nanoparticles to avoid immune detection and clearance.^[^ ^58^ ^]^ This prolongs their circulation time in the body. Additionally, the membrane coating of nanoparticles with NK cell membranes can enhance the immune response against tumors.^[^ ^59^ ^]^ NK cells are known for their ability to secrete various cytotoxic molecules, such as perforin and granzymes, which can induce tumor cell death. By incorporating these molecules into membrane‐coated nanoparticles, they can be delivered directly to the tumor site, leading to enhanced antitumor activity.

**FIGURE 9:**
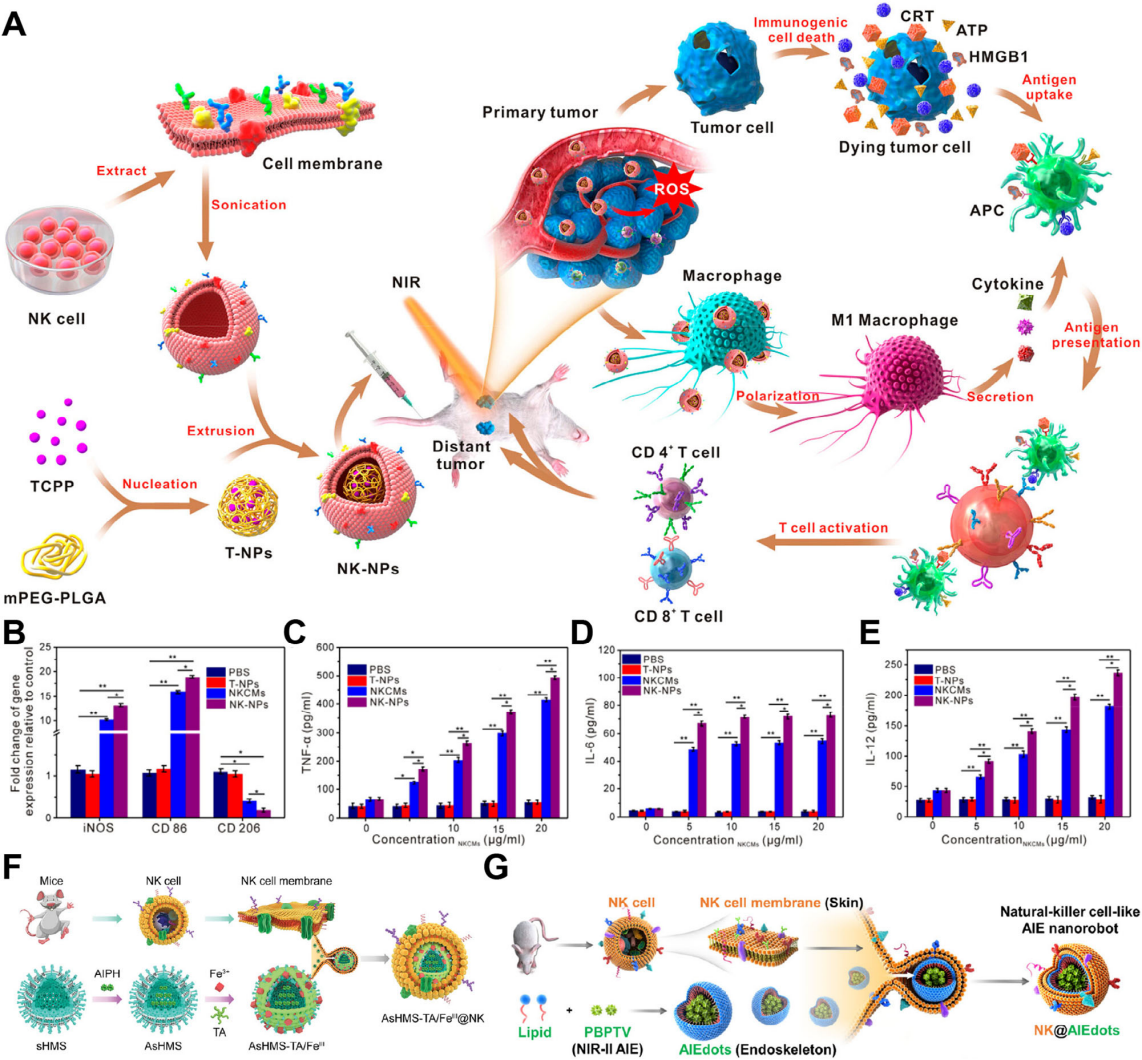
Natural killer cell‐derived membrane‐coated nanoparticles (NK‐NPs) for cancer therapy. (A) Preparation and anticancer mechanism of NK‐NPs. (B) Gene expression patterns associated with M1 macrophage activation in vitro. (C–E) Proinflammatory cytokine levels in THP‐1 cells treated for different durations. Reproduced with permission.^[^ ^56^ ^]^ Copyright 2018, American Chemical Society. (F) Preparation of AsHMS‐TA/Fe^III^@NK. Reproduced under the terms of the CC‐BY 4.0 License.^[^ ^60^ ^]^ Copyright 2021, The Authors, published by Wiley‐VCH GmbH. (G) Preparation of NK@AIEdots. Reproduced with permission.^[^ ^61^ ^]^ Copyright 2020, American Chemical Society.

Taking advantage of the unique characteristics of NK cells, scientists have developed NK‐NPs, which exhibit remarkable efficacy in specifically targeting and eliminating tumor cells. A notable study by Cai et al. introduced an innovative immunotherapy approach using NK cell membrane‐coated nanoparticles loaded with the photosensitizer 4,4′,4′,4′‐(porphine‐5,10,15,20‐tetrayl) tetrakis (benzoic acid) (TCPP), resulting in the formation of NK‐NPs (Figure 9A). Notably, compared with control treatment, treatment with human NK‐NPs led to a substantial increase in the expression of M1 macrophage markers and a decrease in the expression of M2 macrophage markers. These findings indicate that the utilization of NK cell‐derived membranes facilitates the polarization of proinflammatory M1 macrophages induced by human NK‐NPs (Figure 9B). Additionally, the production of M1 macrophage‐related cytokines, including TNF‐α, IL‐6, and IL‐12, was significantly increased (Figure 9C–E). By combining immunogenic PDT with the immune response facilitated by NK cell membranes, NK‐NPs amplify the antitumor immune response.^[^ ^56^ ^]^

Antitumor strategies based on free radicals, such as chemodynamic therapy and thermodynamic therapy, have shown promise in cancer treatment. Chemodynamic therapy utilizes the Fenton/Fenton‐like reaction to convert hydrogen peroxide (H_2_O_2_) into highly reactive hydroxyl radicals (•OH), inducing oxidative stress in tumor cells. However, its efficacy is limited by the acidic pH needed for optimal •OH production, which is challenging to achieve in the TME. To overcome this limitation, Liu et al. developed a multifunctional nanocarrier called AsHMS‐TA/FeIII@NK for efficient tumor targeting and immune evasion (Figure 9F). AsHMS‐TA/FeIII@NK is composed of a virus‐like disulfide‐doped hollow mesoporous silica (sHMS) that encapsulates the thermal azoinitiator 2,2′‐azobis[2‐(2‐imidazolin‐2‐yl) propane] dihydrochloride (AIPH). This nanocarrier was further coated with tannic acid (TA)/Fe^3+^ to enable photothermal assembly. To enhance tumor targeting and immune evasion, the nanocarrier is then cloaked with the NK cell membrane. The use of NK cell membranes in combination with nanoparticle‐based delivery systems holds great potential for enhancing the effectiveness of cancer immunotherapy.^[^ ^60^ ^]^

Nanorobots have emerged as promising tools in nanomedicine, offering improved drug delivery and anticancer efficacy. Researchers developed NK cell‐mimic AIE nanoparticles (NK@AIEdots) by coating an NK cell membrane onto an AIE‐active polymeric nanoendoskeleton (Figure 9G). Biomimetic NK‐cell nanorobots exhibit great potential for active delivery across the blood–brain barrier (BBB) and hold promise for various medical applications.^[^ ^61^ ^]^ In addition to self‐encapsulation around nanoparticles, the NK cell membrane can also be utilized for drug delivery through fusion with liposomes. This approach involves fusing the NK cell membrane with liposomes to create a biomimetic nanoconstruct known as NKsome. The NKsome is formed by isolating an activated NK cell membrane that contains receptor proteins and combining them with fusogenic liposomes. The fusion process allows liposomes to acquire the properties and functionalities of the NK cell membrane. By leveraging the natural targeting abilities of NK cells, NKsome cells exhibit enhanced tumor‐targeting capabilities. The NKsome is loaded with chemotherapeutic drugs, enabling targeted delivery of these drugs to tumor sites. This targeted drug delivery approach enhances the efficacy of treatment while minimizing side effects on healthy tissues.^[^ ^58^ ^]^

#### 4.2.4 DC‐derived membrane‐coated nanoparticles

Dendritic cell‐derived membrane‐coated nanoparticles (DC‐NPs) are a biomimetic nanotechnology platform that involves coating nanoparticles with membranes derived from DCs. By combining the versatility of nanoparticles with the distinctive characteristics of DCs, this approach offers a powerful platform for diverse biomedical applications.^[^ ^62^ ^]^ DCs are a specific category of immune cells renowned for their ability to capture and present antigens to immune cells, thereby instigating targeted immune responses against particular targets. The coating of DC membranes onto nanoparticles allows DC‐NPs to retain the surface markers and functionalities of DCs. Through the interaction of DC‐NPs with immune cells, several specific molecules and molecular interactions emerge as pivotal for shaping their immunological effects.

(1) Major histocompatibility complex (MHC) molecules: Integral to antigen presentation and MHC molecules enable DC‐NPs to present antigens to T cells, thereby initiating targeted immune responses. This process is fundamental for adaptive immunity and the generation of specific immune reactions against encountered antigens.

(2) Costimulatory molecules (e.g., CD80 and CD86): These molecules are crucial for providing secondary signals necessary for T‐cell activation during antigen presentation. DC‐NPs that retain these costimulatory molecules can effectively engage and activate T cells, influencing the magnitude and character of the ensuing immune response.

(3) Toll‐like receptors (TLRs): These cells are abundant in DC membranes, and TLRs play a vital role in recognizing pathogen‐associated molecular patterns (PAMPs) and triggering immune responses. By carrying TLRs, DC‐NPs can effectively engage the immune system, potentially enhancing the immunogenicity of associated antigens and modulating the immune response. These molecules and interactions drive the interplay between DC‐NPs and immune cells, shaping immune responses and demonstrating significant biomedical potential.^[^ ^63^ ^]^ For example, they can be loaded with antigens or immunostimulatory molecules to create vaccines that can specifically target and activate the immune system against certain diseases or pathogens.^[^ ^26^ ^]^

**FIGURE 10:**
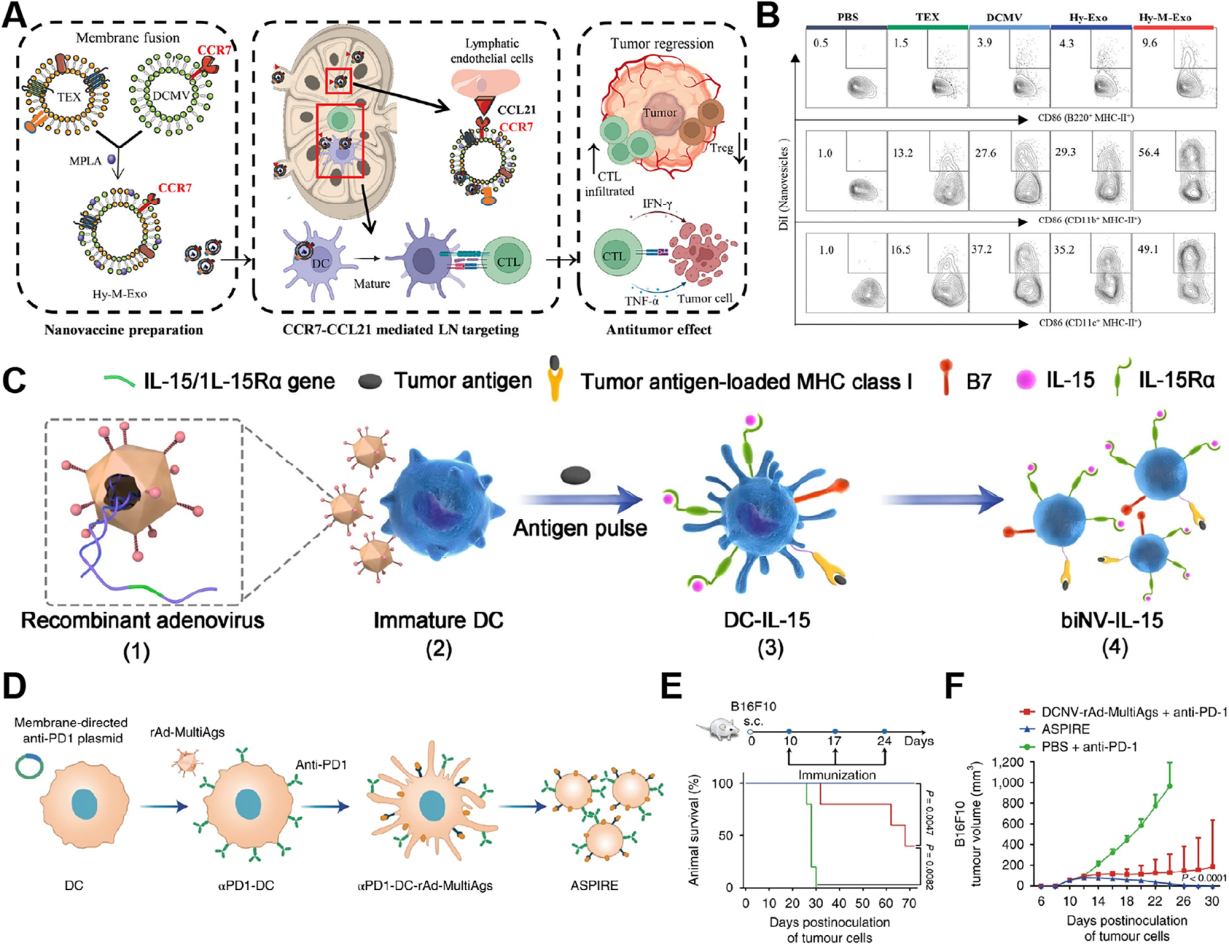
Dendritic cell‐derived membrane‐coated nanoparticles (DC‐NPs) for cancer therapy. (A) Preparation and anticancer mechanism of Hy‐M‐Exos. (B) Flow cytometry diagrams depicting mature antigen‐presenting cells (APCs) in draining lymph nodes (LNs). (*n* = 5). Reproduced with permission. Reproduced under the terms of the CC‐BY 4.0 License.^[^ ^64^ ^]^ Copyright 2023, The Authors, published by Wiley‐VCH GmbH. (C) Preparation of biNV‐IL‐15. Reproducedunder the terms of the CC‐BY License.^[^ ^63b^ ^]^ Copyright 2023, The Authors, published by Springer Nature. (D) Preparation of ASPIRE. (E,F) Survival and tumor growth curves were generated for the different treatment groups. (*n* = 5). Reproduced with permission.^[^ ^65^ ^]^ Copyright 2022, Springer Nature.

Therapeutic vaccination by stimulating the immune system has shown promise as a cancer treatment strategy. However, the lack of well‐defined tumor‐associated antigens (TAAs) in spontaneous tumors, such as head and neck squamous cell carcinoma (HNSCC), presents a challenge for antigen and adjuvant‐based vaccine development. To overcome this challenge, Liang et al. developed a novel nanovaccine called Hy‐M‐Exo by combining tumor‐derived exosomes (TEXs) with dendritic cell membrane vesicles and adjuvanted it with monophosphoryl lipid A (MPLA), a toll‐like receptor 4 (TLR4) adjuvant (Figure 10A). The Hy‐M‐Exo nanovaccine specifically targets lymph nodes (LNs) via lymph drainage, inducing a robust immune response while minimizing systemic toxicity. The Hy‐M‐Exo nanovaccine exhibited protein expression similar to that of DCs, including that of CD86 and CCR7, and effectively targeted the paracortex of LNs. In preclinical studies, Hy‐M‐Exos were found to stimulate robust activation of B cells, macrophages, and DCs in LNs, leading to a significant innate immune response (Figure 10B). Additionally, Hy‐M‐Exos were observed to downregulate regulatory T cells, thereby reducing immune tolerance.^[^ ^64^ ^]^

In addition to their inherent properties, DC‐NPs can also be surface modified to further enhance their functionality and effectiveness. Surface modification allows the introduction of specific molecules or functionalities onto the DC‐NP outer membrane, expanding its capabilities and enabling targeted interactions with the immune system. Cytokine therapy, specifically using interleukin‐15 (IL‐15), has demonstrated significant potential. However, the clinical use of IL‐15 has been restricted by factors such as severe toxicity, low immune response rates, systemic immune activation, and short half‐life. To address these challenges, Sun et al. developed a biomimetic nanovaccine called biNV‐IL‐15, which incorporates multivalent IL‐15 self‐presentation. This nanovaccine is derived from genetically engineered DCs and simultaneously immobilizes IL‐15/IL‐15 receptor α (IL‐15Rα), TAA peptide/major histocompatibility complex class I (MHC‐I) complexes, and relevant costimulatory molecules (Figure 10C). BiNV‐IL‐15 has several advantages: (1) its molecular components closely resemble those of living DCs, including the expression of major histocompatibility complex (MHC) I/antigenic peptide complexes and costimulatory molecules; (2) as a cell‐free approach, it mitigates the risks associated with cellular therapies, such as in vivo replication; and (3) its appropriate size (110 nm) and abundant surface membrane receptors facilitate extensive distribution in lymph nodes.^[^ ^63b^ ^]^

In addition to delivering IL‐15, DCs can also simultaneously deliver anti‐PD‐1 agents and antigens for antitumor purposes. Liu et al. presented a novel nanovaccine formulation called ASPIRE (antigen self‐presentation and immunosuppression reversal) for personalized cancer immunotherapy (Figure 10D). The ASPIRE nanovaccine utilizes artificial cytomembrane nanovesicles derived from DCs to achieve targeted presentation of antigen epitopes through MHC‐I molecules. It also incorporates the delivery of anti‐PD‐1 agents through genetic engineering. The ASPIRE nanovaccine synergistically combines these two elements to boost the immune response against cancer. The MHC‐I molecules on nanovesicles present specific antigen epitopes, leading to the activation of CD8^+^ T cells, which play a vital role in eradicating tumor cells. Simultaneously, the delivered anti‐PD‐1 antibody blocks the PD‐1 pathway, preventing the suppression of T‐cell function and allowing for a more effective antitumor immune response. The research findings demonstrated that mice receiving ASPIRE treatment exhibited significant tumor suppression and extended survival (Figure 10E,F).^[^ ^65^ ^]^

Overall, DC‐NPs offer a promising platform for targeted immunotherapy, vaccine development, and immune modulation. By leveraging the unique properties of DCs, DC‐NPs hold great potential for advancing personalized medicine and improving immune‐related treatments.

### 4.3 Cancer cell‐derived membrane‐coated nanoparticles

Cancer cell‐derived membrane‐coated nanoparticles (CM‐NPs) combine the advantages of cancer cell membranes and nanoparticles for targeted drug delivery.^[^ ^66^ ^]^ Synthetic nanoparticles are coated with cancer cell membranes, allowing specific binding to cancer cells and improving the accumulation of therapeutic agents at the tumor site. CM‐NPs can effectively target cancer cells through a mechanism known as homotypic targeting.^[^ ^67^ ^]^ This process relies on the recognition of specific surface markers or antigens present on the cancer cell membrane by complementary receptors on the nanoparticle surface. The homotypic targeting mechanism of CM‐NPs relies on specific molecular interactions between surface molecules. For instance, one prominent molecule involved in this process is the integrin family of cell adhesion receptors. These integrins, such as αvβ3 integrin, are often overexpressed on the surface of cancer cells. When CM‐NPs carry these integrins, they can effectively bind to other integrins of the same type on the cancer cell membrane, facilitating targeted delivery.^[^ ^68^ ^]^ Another set of molecules crucial in this process are selectins, which are involved in cell adhesion and are often upregulated in cancer cells.^[^ ^69^ ^]^ By incorporating selectins from cancer cells into the membrane coating of nanoparticles, these particles can mimic the adhesive properties of cancer cells, enhancing their ability to target specific tissues or organs.^[^ ^5d^ ^]^ Additionally, the presence of specific glycoproteins, such as mucins and glycosylated proteins, on the cancer cell membrane plays a significant role in the targeting mechanism.^[^ ^70^ ^]^ These glycoproteins can be mirrored on the surface of nanoparticles, allowing them to exploit the unique glycosylation patterns found on cancer cells for precise targeting. Overall, the incorporation of these and other specific molecules from the cancer cell membrane onto the surface of nanoparticles enables homotypic targeting, ensuring that the nanoparticles effectively recognize and bind to cancer cells through specific molecular interactions. By utilizing this targeted delivery approach, off‐target effects are minimized, and the effectiveness of the treatment is increased.^[^ ^71^ ^]^ The membrane coating enhances biocompatibility by mimicking cancer cell membranes, reducing immune recognition and potential immune responses. It also acts as a stealth camouflage, prolonging circulation time in the bloodstream and increasing the chances of nanoparticles reaching the tumor site.^[^ ^26^ ^]^ CM‐NPs loaded with anticancer drugs, imaging agents, or genetic material hold great promise in personalized medicine and precision oncology, as they specifically target cancer cells while minimizing off‐target effects.^[^ ^72^ ^]^

**FIGURE 11:**
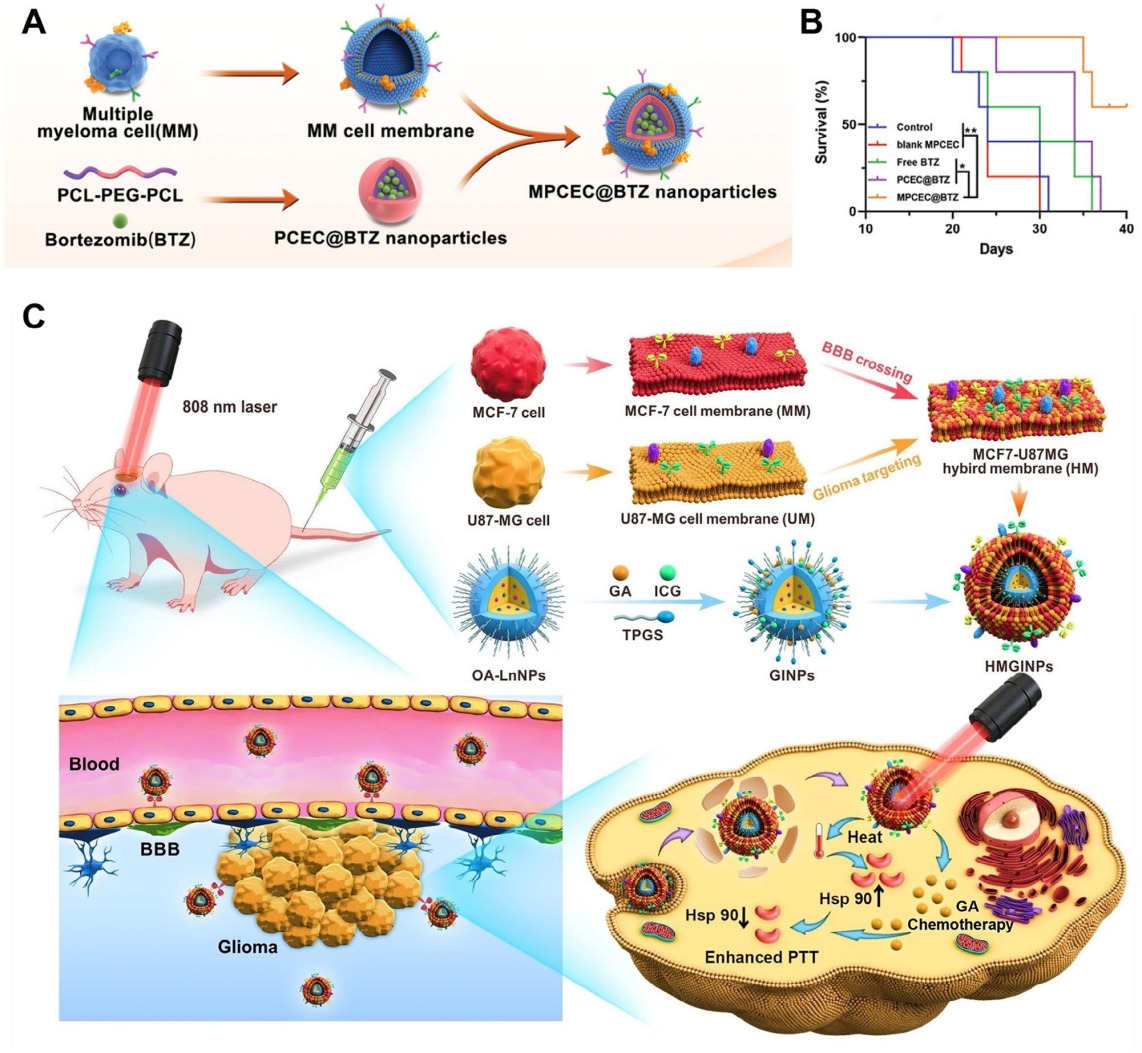
Cancer cell‐derived membrane‐coated nanoparticles (CM‐NPs) for cancer therapy. (A) Preparation of MPCEC@BTZ. (B) Survival curves of mice after different treatments. Reproduced with permission.^[^ ^73^ ^]^ Copyright 2022, Wiley‐VCH. (C) Preparation and anticancer mechanism of HMGINPs. Reproduced with permission.^[^ ^74^ ^]^ Copyright 2023, Wiley‐VCH.

Multiple myeloma (MM) is a type of hematological cancer characterized by the abnormal proliferation of plasma cells in the bone marrow (BM). The curative potential of the current therapeutic approaches for multiple myeloma (MM) is limited, primarily due to the challenges associated with delivering a sufficient amount of drugs to the BM. This difficulty in drug delivery hampers the eradication of MM cells. To address this challenge, Qian et al. developed an MM‐mimicking nanocarrier by encapsulating bortezomib (BTZ) within poly(ε‐caprolactone)‐poly(ethylene glycol)‐poly(ε‐caprolactone) (PCEC) nanoparticles and coating them with the cell membrane of MM cells. (Figure 11A). This nanocarrier takes advantage of the homing characteristic of MM cells to the BM, which is primarily influenced by surface molecules on the tumor cell membrane. By mimicking MM cells, MM‐mimicking nanoparticles can enter the BM as a “Trojan horse” and target tumor cells through homologous targeting. This approach enhances drug availability at the myeloma site, effectively inhibiting MM growth. Additionally, MM‐mimicking nanoparticles have the ability to escape phagocytosis by the mononuclear phagocyte system, leading to prolonged circulation. In vivo therapeutic experiments have demonstrated excellent treatment efficacy for MM using this strategy (Figure 11B).^[^ ^73^ ^]^

Glioma, a highly aggressive form of brain tumor, is associated with a high fatality rate and short survival time. Its rapid growth and invasive nature contribute to its challenging treatment. Timely intervention is crucial, particularly in early‐stage glioma. However, the blood‒brain barrier (BBB) poses a significant obstacle by restricting the entry of therapeutic agents into the brain. Moreover, the nonspecific distribution of these agents often leads to adverse effects on delicate cerebral tissues. Additionally, the nontargeted distribution of these agents often leads to side effects on vulnerable cerebral tissues. To address the abovementioned challenges, Liu et al. proposed a hybrid cell membrane (HM) camouflage strategy. This innovative approach involves combining the cell membranes of brain metastatic breast cancer (MCF‐7) cells and glioma (U87‐MG) cells to create HMs. By employing this method, the resulting HM acquires both the ability to cross the BBB and the homologous tumor‐targeting ability of the two source cell membranes. Homologous tumor targeting is achieved by coating nanocomposites with source cancer cell membranes, providing a more precise and effective approach than active tumor targeting based on single ligand‒receptor interactions. This is because the homologous targeting process relies on the presence of various functional proteins on the biomimetic membranes. Researchers have constructed HMGINPs by applying HM coatings to nanoparticles loaded with gambogic acid (GA) and indocyanine green (ICG). These nanoparticles, also known as GINPs, serve as delivery systems for therapeutic agents (Figure 11C). With their ability to cross the BBB, HMGINPs effectively deliver drugs into the brain, while their homologous glioma‐targeting ability ensures precise accumulation in glioma cells. This targeted approach minimizes the potential toxic side effects on healthy brain tissues. To further enhance the therapeutic effect of GA, researchers have loaded HMGINPs with GA, an inhibitor of heat shock proteins (HSPs), and ICG, a photothermal agent. This combination increases the thermal sensitivity of glioma cells, enabling more effective PTT. Importantly, this study is the first to explore the use of a hybrid cell membrane derived from the brain metastatic cancer cell membrane and glioma cell membrane for simultaneous BBB penetration and glioma targeting.^[^ ^74^ ^]^

In addition to fusing different types of cancer cell membranes, the fusion of mitochondrial membranes with tumor cell membranes can also be explored as a potential approach. Shi et al. developed a hybrid membrane‐camouflaged nanoparticle called HM‐NPs@G, which involved fusing cancer cells and mitochondrial membranes. This innovative technique enabled the encapsulation of Gboxin‐loaded nanoparticles (NPs@G) within the hybrid membrane, facilitating the specific delivery of Gboxin to the mitochondria of GBM cells. Through this innovative approach, they aimed to enhance the effectiveness of Gboxin by specifically delivering it to mitochondria, where it can induce apoptosis in tumor cells. This targeted delivery system shows immense potential in enhancing the treatment outcomes of GBM.^[^ ^75^ ^]^

### 4.4 Platelet‐derived membrane‐coated nanoparticles

Platelet‐derived membrane‐coated nanoparticles (PM‐NPs) are a type of nanoscale drug delivery system that involves coating nanoparticles with membranes derived from platelets.^[^ ^76^ ^]^ Platelets, which are blood cells involved in wound healing and clotting processes, possess unique membrane properties that make them highly suitable for targeted drug delivery applications. They tend to accumulate at sites of inflammation, injury, and tumor formation. The specific mechanism underlying this phenomenon involves the interaction of platelets with the endothelial cells lining blood vessels. In response to injury or inflammation, these endothelial cells undergo changes, leading to exposure to von Willebrand factor (vWF) and other adhesion molecules. Platelets, in turn, have receptors such as glycoprotein Ib (GPIb) and glycoprotein IIb/IIIa (GPIIb/IIIa) that can bind to vWF and other adhesion molecules exposed by activated endothelial cells.^[^ ^77^ ^]^ This interaction triggers the adhesion and subsequent activation of platelets at the site of injury or inflammation. Moreover, platelets also express P‐selectin on their surface upon activation. P‐selectin plays a crucial role in platelet‐leukocyte interactions, further contributing to the recruitment of immune cells to sites of injury or inflammation.^[^ ^78^ ^]^ Given these mechanisms, the unique membrane properties of platelets, along with their tendency to accumulate at sites of injury, inflammation, and tumor formation, make them attractive vehicles for targeted drug delivery, particularly in the context of these specific pathological conditions. By utilizing platelet‐derived membranes, PM‐NPs can effectively mimic the natural targeting ability of platelets, enabling them to specifically target disease sites, including tumors, and deliver therapeutic agents directly to the affected area.^[^ ^79^ ^]^

**FIGURE 12:**
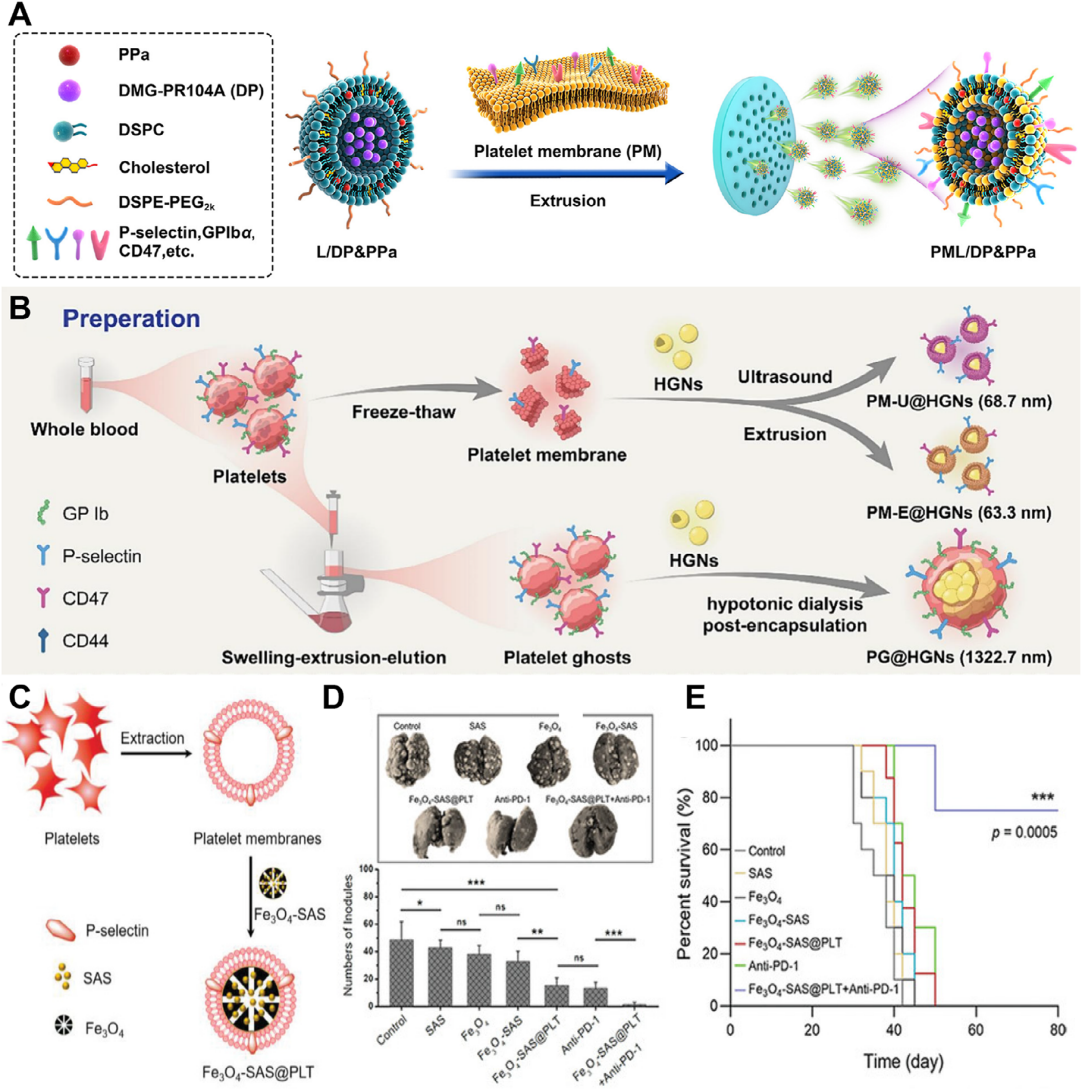
Platelet‐derived membrane‐coated nanoparticles (PM‐NPs) for cancer therapy. (A) Preparation of PML/DP&PPa. Reproduced with permission.^[^ ^80^ ^]^ Copyright 2022, Elsevier. (B) Preparation of PG@HGNs. Reproduced with permission.^[^ ^81^ ^]^ Copyright 2022, Elsevier. (C) Preparation of Fe_3_O_4_‐SAS@PLT. (D) The number of lung metastasis nodules and representative lung photographs were assessed for the different groups (*n* = 5). (E) Survival curves of mice in different groups (*n* = 10). Reproduced with permission.^[^ ^82^ ^]^ Copyright 2020, Wiley‐VCH.

The TME possesses unique characteristics that can be utilized for developing effective antitumor nanomedicine strategies. However, the heterogeneity of tumors often limits therapeutic outcomes. To overcome this challenge, researchers can exploit the amplifying characteristics of the TME, which are regulated by vascular disrupting agents (VDAs), to enhance the efficacy of nanomedicines. Sun et al. prepared a conjugate of N,N‐dimethylglycine (DMG)‐PR104A (DP) and coaded it with PR104A‐pyropheophorbide a (PPa) in liposomes (L/DP&PPa). Subsequently, they fabricated biomimetic liposomes called platelet membrane‐fusogenic liposomes (PML/DP&PPa), also referred to as “platesomes,” by fusing platelet membranes with L/DP&PPa (Figure 12A). This combined strategy involving an artificially regulated TME and bioinspired platesomes significantly improved tumor drug delivery and enhanced hypoxia‐selective tumor activation. This approach provides a promising solution for achieving highly efficient cancer therapy.^[^ ^80^ ^]^

The widespread presence of vascular endothelial gaps in normal tissues often leads to off‐target accumulation of nanoscale drug delivery systems. To overcome this issue, Zhang et al. employed microsized cellular platelet “ghosts” (PGs) as carriers to transport hollow gold nanoparticles (HGNs), creating a hierarchical biosafe system called PG@HGNs (Figure 12B). The preparation of PGs involved an optimized method called the “swelling‐extrusion‐elution” technique, while the loading of HGNs into PGs was achieved using a “hypotonic dialysis” method. In mouse models, the PG@HGNs demonstrated prolonged circulation, likely attributed to the presence of the CD47 “self‐recognition” receptor on the PGs. Moreover, their microscale size effectively reduced the interception of normal tissues, further enhancing the tumor‐targeting efficiency of the HGNs.^[^ ^81^ ^]^

Combining photoimmunotherapy, such as PDT or PTT, with immunotherapy has the potential to boost the immune response. However, excessive immunogenicity can result in the development of severe inflammatory reactions. To address this issue, Yang et al. proposed a novel approach that integrates PD‐1 immune checkpoint blockade therapy with the induction of ferroptosis using biomimetic nanoparticles. In their strategy, they employ sulfasalazine (SAS), a medication commonly used for treating rheumatoid arthritis. SAS not only inhibits the migration of inflammatory cells and the IκB kinase pathway but also suppresses tumor growth. Additionally, SAS induces ferroptosis by limiting the cellular uptake of cysteine. This innovative approach offers a potential solution for mitigating excessive immunogenicity while enhancing therapeutic efficacy. To enhance the effectiveness of SAS and reduce its dosage, they loaded SAS into magnetic nanoparticles (Fe_3_O_4_) and cumulatively mixed them with platelet membranes (Fe_3_O_4_‐SAS@PLT) for tumor ferroptosis treatment (Figure 12C). The experimental findings demonstrated that mice treated with Fe_3_O_4_‐SAS@PLT in combination with anti‐PD‐1 therapy exhibited remarkable suppression of tumor metastasis. In the lungs, only a minimal number of metastatic nodules were observed (Figure 12D). Notably, the survival rate of the mice in this treatment group reached 76% at 80 days, in stark contrast to all the mice in the other groups, which died within 50 days (Figure 12E). The utilization of PM‐NPs not only enhances immune evasion but also enhances tumor targeting, facilitating the optimal delivery of ferroptosis‐inducing nanoparticles to the tumor site. This innovative approach holds significant potential for improving treatment outcomes and prolonging survival in cancer patients.^[^ ^82^ ^]^

In summary, CMNPs derived from different cell sources offer distinct advantages in cancer therapy. Their applications in drug delivery, imaging, and combination therapy have demonstrated significant potential for enhancing treatment efficacy, reducing systemic toxicity, and improving patient outcomes. Further research and development could unveil more efficacious and personalized strategies for combating cancer.

## 5 CONCLUSIONS AND FUTURE PERSPECTIVES

CMNPs have emerged as a highly promising approach for achieving targeted drug delivery in cancer therapy. This approach provides improved biocompatibility, stability, and targeting capabilities. Various cell membrane sources, including RBCs, immune cells, cancer cells, and platelets, have been utilized in this technology. In the field of cancer treatment, CMNPs have shown tremendous potential. By selecting specific cell membrane sources, targeted therapy for tumors can be achieved. These nanoparticles can deliver drugs, genes, or immunomodulators, enabling a multifaceted attack on tumors and improving treatment outcomes.

However, CMNPs are several challenges that need to be addressed before CMNPs can be widely adopted in clinical settings. One major challenge is the difficulty in achieving large‐scale production of these nanoparticles. The production process needs to be scalable to meet the demands of clinical studies, which is currently a significant hurdle. Another challenge lies in understanding the biological foundations of the cell membrane and its mechanisms of action. Despite the therapeutic potential of CMNPs demonstrated in numerous preclinical studies, the lack of comprehensive understanding of the underlying mechanisms of CMNPs hinders their translation into clinical application. Furthermore, the reproducibility of studies using CMNPs is crucial. Variations between different batches of CMNPs need to be carefully controlled to ensure consistent and reliable results. This requires the development of standardized protocols and quality control measures for the production and characterization of nanoparticles.

Future research can focus on the following aspects: (1) long‐term stability and biological degradation: investigate CMNPs’ long‐term stability, biological degradation, and potential accumulation in off‐target organs or tissues to ensure their safety and minimize unforeseen adverse effects; (2) immunogenicity and immunomodulatory effects: explore how CMNPs interact with the immune system, including their impact on immune responses and long‐term immunological safety; (3) clinical translation and regulatory approval: develop standardized guidelines for evaluating CMNPs clinically, including methodologies for assessing safety, efficacy, and quality control, to facilitate regulatory approval and clinical integration; (4) targeting precision and therapeutic efficacy: investigate factors influencing CMNP targeting efficiency to optimize therapeutic efficacy and minimize off‐target effects in diverse clinical scenarios; and (5) multifunctional CMNPs and combination therapies: explore engineering multifunctional CMNPs and combining them with other therapies to enhance treatment outcomes for complex disease states.

In conclusion, the application of CMNPs in cancer treatment shows great potential. Continued research and development in this field will further advance our knowledge and utilization of these nanoparticles, leading to the creation of improved and tailored treatment approaches for individuals with cancer. This ongoing exploration will undoubtedly contribute to the development of more efficient and personalized strategies for the management of cancer patients.

## CONFLICT OF INTEREST STATEMENT

The authors declare no competing interests. Xiaoyuan Ji is a member of the *Exploration* editorial board, and he was not involved in the handling or peer review process of this manuscript.
